# Supplementary material for: Dynamic Runx1 chromatin boundaries affect gene expression in hematopoietic development
Source: Nat Commun. 2022 Feb 9;13:773. doi: 10.1038/s41467-022-28376-8 (PMC8828719; doi:10.1038/s41467-022-28376-8)
Supplement: Supplementary file 1 — Supplementary Information [file 41467_2022_28376_MOESM1_ESM.pdf]

Supplementary Information for

Dynamic *Runx1* chromatin boundaries affect gene expression in hematopoietic development

Owens *et al*.

Supplementary Figure 1.

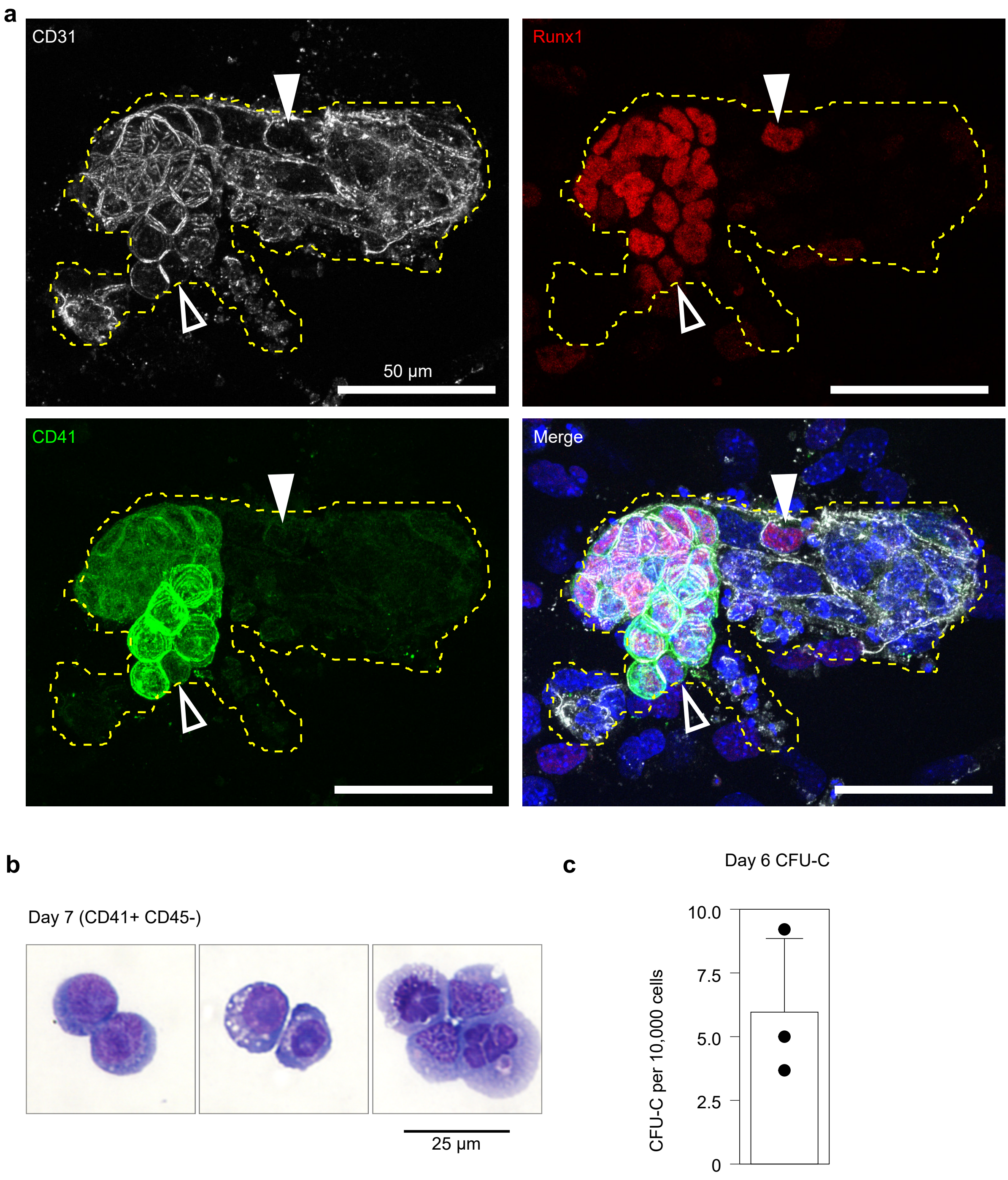

Supplemetary Figure 1 – *In vitro* hematopoietic differentiation mimics endothelial-to-hematopoietic transition *in vivo*. **a**) Confocal imaging (maximum intensity projections) of immunocytochemical staining of day 7 cultures with CD31 shown in white, Runx1 in red, CD41 in green, and DAPI in blue. A colony of hemogenic endothelial (HE) cells and emerging hematopoietic progenitors is outlined by the yellow dashed line. A Runx1+CD31+CD41-/lo HE cell is indicated by the solid white arrowhead. Emerging hematopoietic progenitors are indicated by the hollow white arrowhead. Representative images are shown. Experiments were performed more than five times with similar results. **b**) May-Grünwald staining of cytospins of FACS-isolated CD41+ CD45- cells. Representative images are shown. Experiments were performed more than three times with similar results. **c**) Hematopoietic colony formation assays (CFU-C) on day 6 whole cultures. The bar shows the mean number of colonies formed per 10,000 cells, and error bar represents standard deviation. Data were analyzed from n=3 biologically independent experiments. Source data are provided as a Source Data file.

Supplementary Figure 2.

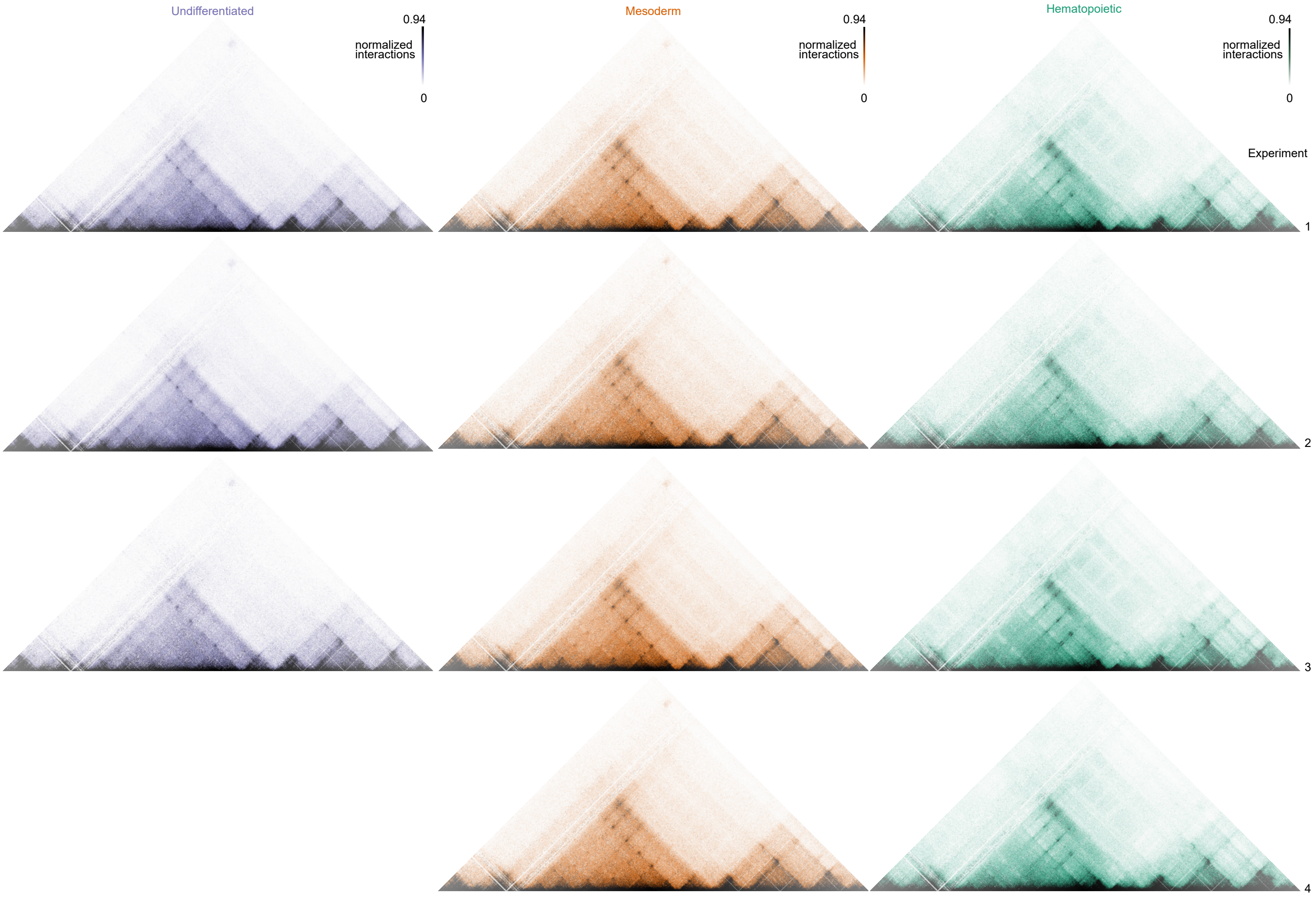

Supplemetary Figure 2 – Tiled-C matrices of individual replicates in wild type cells over hematopoietic differentiation. Tiled-C matrices from individual experiments are shown at 2 kb resolution and are total count and ICE normalized. All matrices are visualized with a threshold set at the 94th percentile.

Supplementary Figure 3.

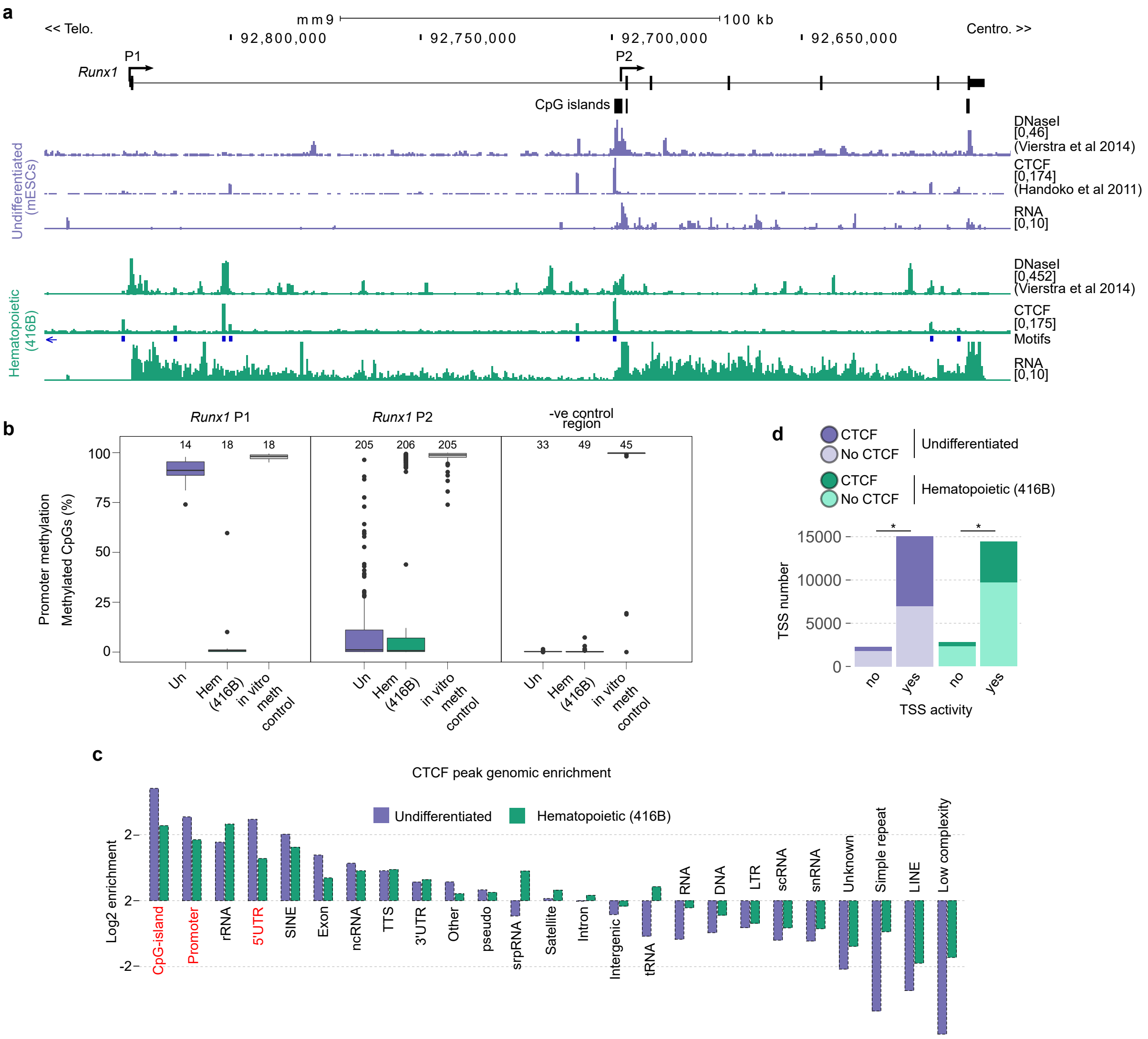

Supplemetary Figure 3 – CTCF binding close to *Runx1* promoters and promoters genome wide is associated with promoter activity. **a**) *Runx1* locus showing chromatin marks and poly(A)-minus RNA-seq in undifferentiated mESCs and 416B cells. Public data that were reanalyzed include mESC and 416B DNaseI[1] and mESC CTCF[2]. The orientation of CTCF motifs are indicated underneath peaks. **b**) Analysis of *Runx1* promoter methylation by targeted bisulfite sequencing of undifferentiated mESCs, hematopoietic 416B cells. *In vitro* methylated DNA and an additional negative control site are shown as controls. Boxplot centre shows median, bounds of the box indicate 25th and 75th percentiles, and maxima and minima show the largest point above or below 1.5 * interquartile range. Data were analyzed from the total number of CpG dinucleotides indicated above each boxplot from one experiment. **c**) Enrichment of CTCF peaks in undifferentiated mESCs and 416B cells at genomic features annotated using HOMER (4.7) annotatePeaks.pl. **d**) Association between CTCF binding within 5 kb of transcription start sites (TSS) shown for active and inactive TSSs in undifferentiated mESCs and 416B hematopoietic cells. CTCF ChIP-seq in mESC was reanalyzed from previously published data[2]. Pearson's one-tailed Chi-squared test with Yates' continuity correction was performed with p-values adjusted for multiple hypothesis testing using the Holm method: Undifferentiated (*, p = 1.29×10^{-185}), hematopoietic, 416B (*, p = 1.49×10^{-68}).

Supplementary Figure 4.

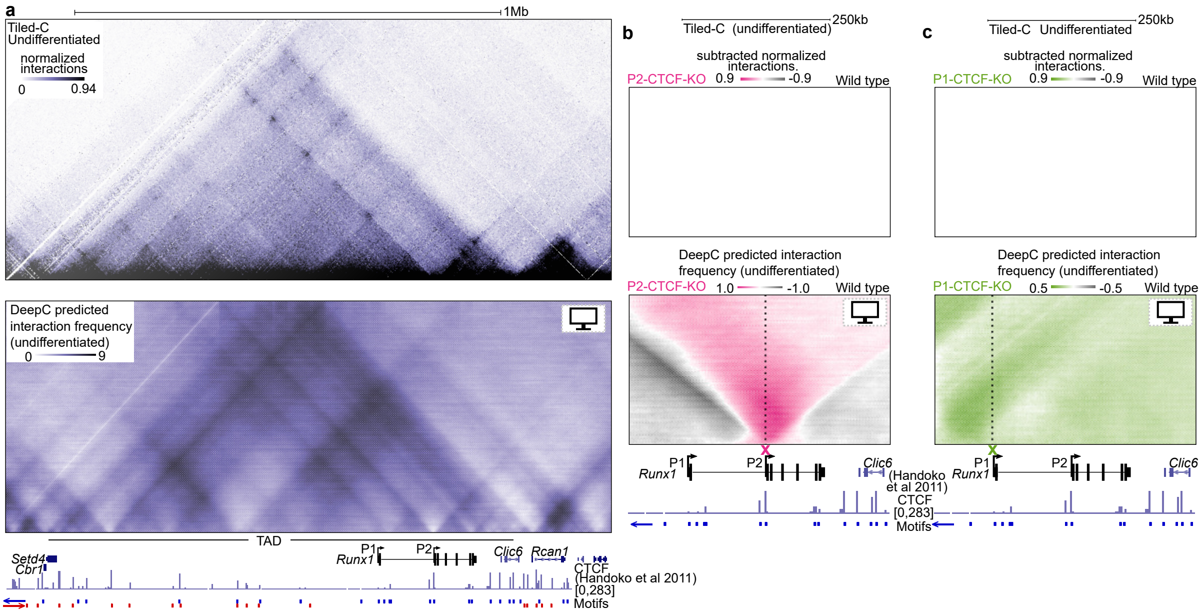

Supplemetary Figure 4 – DeepC predictions of *Runx1* chromatin interactions and promoter-proximal CTCF site deletions in undifferentiated mESCs. **a**) Tiled-C (top panel) and deepC prediction of chromatin interactions (bottom panel) at the *Runx1* locus in undifferentiated mESCs. The overall 1.1Mb *Runx1* TAD indicated below the matrices was substantially concordant between the deepC predictions and real Tiled-C data. CTCF occupancy[2] and motif orientation are indicated. **b, c**) Tiled-C (top panels) and deepC predictions (bottom panels) of chromatin interactions after P2-CTCF (B) and P1-CTCF (C) deletion. DeepC predicted that, in undifferentiated mESCs, P2-CTCF deletion would lead to increased interactions across the boundary (B, bottom panel, pink upside down triangle with its tip at the P2-CTCF site) and a decrease in a stripe of interactions emanating from the CTCF site (B, bottom panel, dark grey line at 45°). Both features are visible in Tiled-C data in undifferentiated mESCs (B, top panel). Little change in interactions was seen after loss of P1-CTCF in mESC (C, top panel), which was similar to the prediction by deepC (C, bottom panel). CTCF binding[2] and motif orientation are indicated below the matrices.

Supplementary Figure 5.

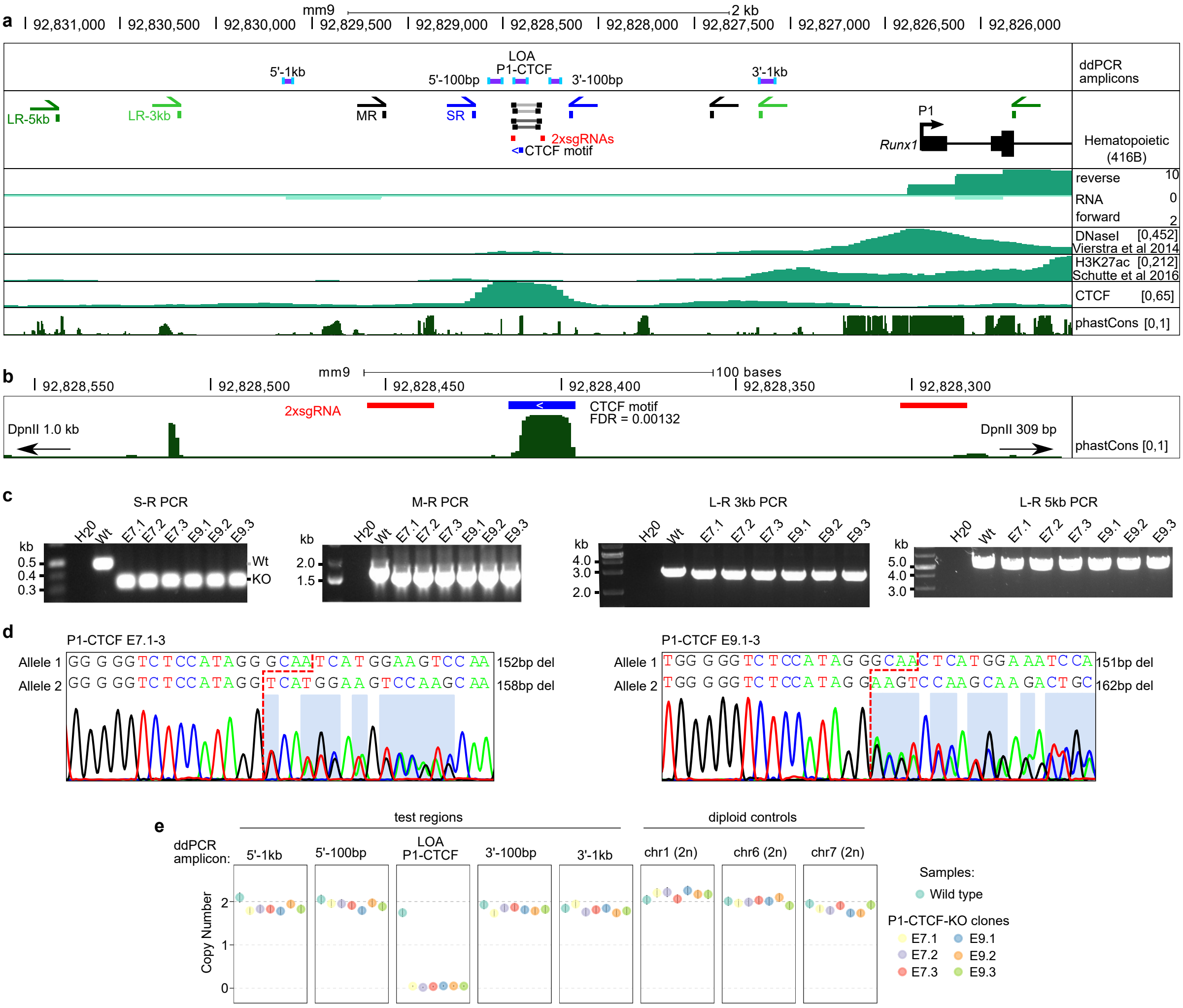

Supplemetary Figure 5 – Strategy for the generation of P1-CTCF knock-out clones using CRISPR/Cas9. a) Schematic of the *Runx1* P1 promoter and upstream promoter-proximal CTCF site with single guide RNAs (sgRNAs), short-range (SR), medium range (MR), longer range 3 kb (LR-3kb) and longer range 5 kb (LR-5kb) PCR primer locations indicated. The de novo CTCF motif identified under the CTCF peak (P1-CTCF) is indicated with its orientation. Droplet digital PCR (ddPCR) amplicon positions are indicated and were spaced upstream (5') or downstream (3') of sgRNA cut positions by approximately the indicated distances. One ddPCR amplicon was placed between sgRNA cut sites to detect loss of the CTCF motif (LOA, loss of allele). Poly(A) minus RNA-seq and CTCF ChIP-seq tracks for 416B cells are included. Publicly available DNaseI[1] and H3K27ac ChIP-seq[3] data for 416B cells were reanalyzed and shown in the respective tracks. Evolutionary sequence conservation shown is vertebrate conservation by PhastCons[4]. **b**) Close-up view of dual sgRNA (2xsgRNA) targeting strategy to delete a conserved CTCF motif using CRISPR/Cas9. The location of the nearest DpnII restriction sites is indicated and were not disrupted by targeting. **c**) Gel electrophoresis images of PCR samples from six independent sub-clones derived from two separate targeting events. Source data are provided as a Source Data file. **d**) Base calls from sequencing of sub-clones revealing homozygous deletion of the CTCF motif. **e**) Copy number analysis by ddPCR across the targeted region (test amplicons are indicated on the locus schematic in (a)) and on three diploid (2n) control chromosomes. Test amplicons were designed to cover the critical region containing P1-CTCF (LOA, loss of allele), and spanning in the 5' and 3' direction from the targeted site at approximately 100 bp and 1 kb to detect larger deletions. Each ddPCR reaction contained an additional internal control amplicon on chromosome 4 (which was not targeted in this study, see methods) to facilitate interpretation of deleted regions such as P1-CTCF. Each dot represents the relative copy number of a sample over the indicated test amplicon, normalized to three diploid control regions with 95% confidence intervals calculated from the Poisson distribution indicated. Source data are provided as a Source Data file.

Supplementary Figure 6.

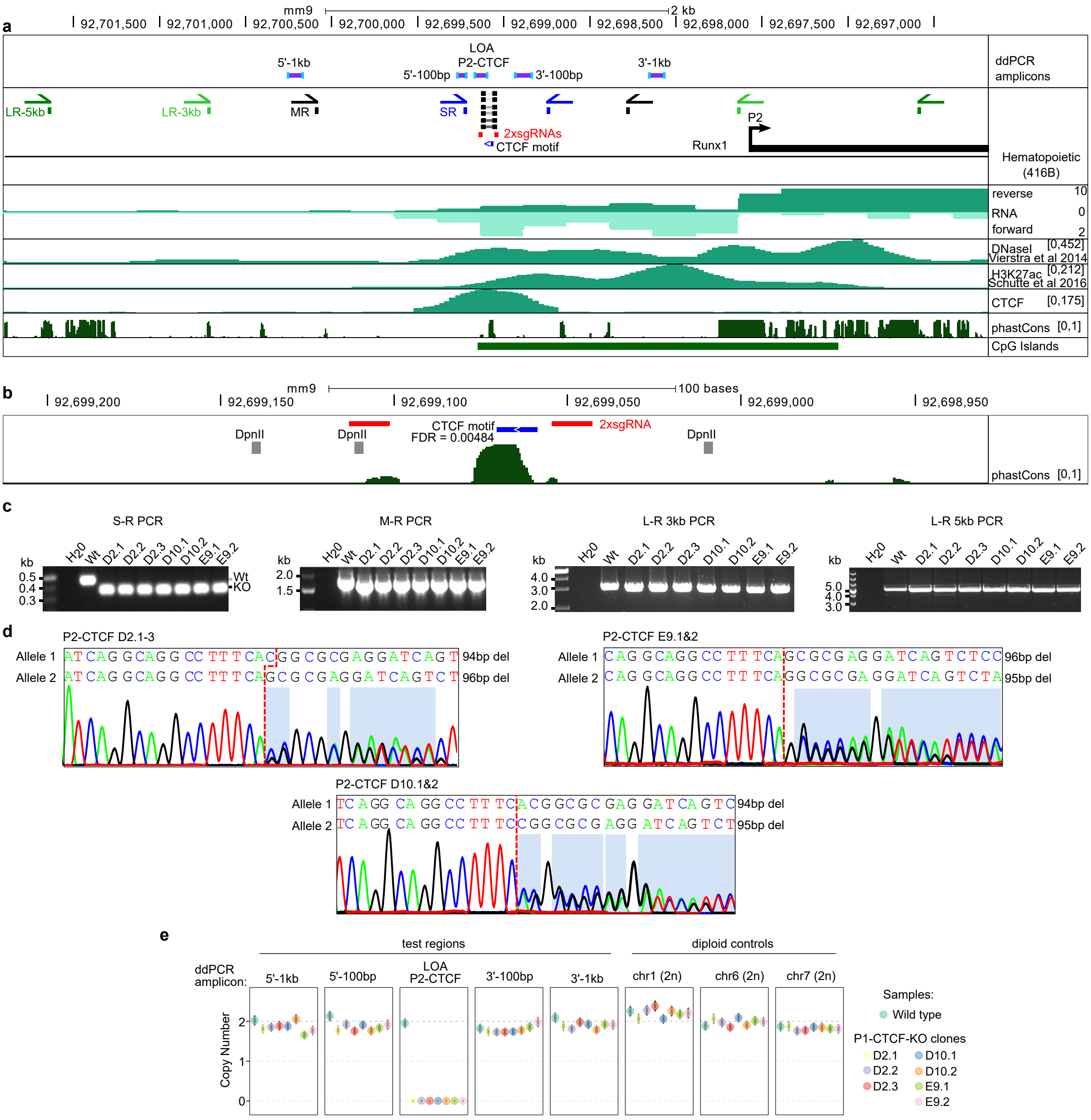

Supplemetary Figure 6 – Strategy for the generation of P2-CTCF knock-out clones using CRISPR/Cas9.a) Schematic of the *Runx1* P2 promoter and adjacent upstream promoter-proximal CTCF site with single guide RNAs (sgRNAs), short-range (SR), medium range (MR), longer-range 3 kb (LR-3kb) and longer range-5 kb (LR-5kb) PCR primer locations indicated. The de novo CTCF motif identified under the CTCF peak (P2-CTCF) is indicated with its orientation. Droplet digital PCR (ddPCR) amplicon positions are indicated and were spaced upstream (5') or downstream (3') of sgRNA cut positions by approximately the indicated distances. One ddPCR amplicon was placed in between sgRNA cut sites to detect loss of the CTCF motif (LOA, loss of allele). Poly-A minus RNA-seq and CTCF tracks for 416B cells are included. Publicly available DNaseI[1] and H3K27ac ChIP-seq[3] data for 416B cells were reanalyzed and shown in the respective tracks. CpG island annotations are from UCSC genome browser[5]. Evolutionary sequence conservation shown is vertebrate conservation by PhastCons[4]. **b**) Close-up view of dual sgRNA (2xsgRNA) targeting strategy to delete a conserved CTCF motif using CRISPR/Cas9. The location of the nearest DpnII restriction sites is indicated and were not disrupted by targeting. **c**) Gel electrophoresis images of PCR samples from seven independent sub-clones derived from three separate targeting events. Source data are provided as a Source Data file. **d**) Base calls from sequencing of sub-clones revealing homozygous deletion of the CTCF motif. **e**) Copy number analysis by ddPCR across the targeted region (test amplicons are indicated on the locus schematic in (a)) and on three diploid (2n) control chromosomes. Test amplicons were designed to cover the critical region containing P2-CTCF (LOA, loss of allele), and spanning in the 5' and 3' direction from the targeted site at approximately 100 bp and 1 kb to detect larger deletions. Each ddPCR reaction contained an additional internal control amplicon on chromosome 4 (which was not targeted in this study, see methods) to facilitate interpretation of deleted regions such as P2-CTCF. Each dot represents the relative copy number of a sample over the indicated test amplicon, normalized to three diploid control regions, with 95% confidence intervals calculated from the Poisson distribution indicated. Source data are provided as a Source Data file.

Supplementary Figure 7.

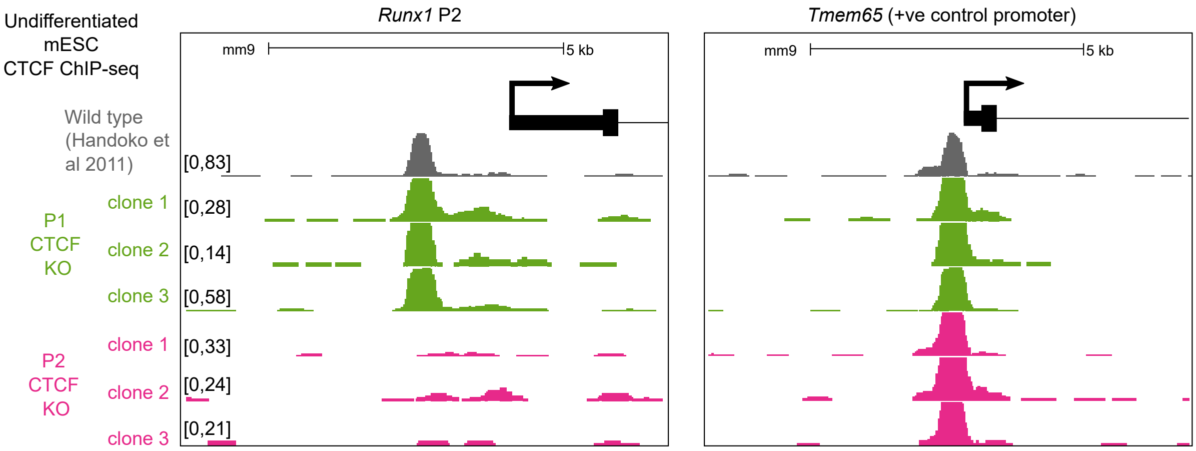

Supplemetary Figure 7 – Loss of CTCF binding at P2-CTCF in P2-CTCF-KO clones.CTCF ChIP-seq in six undifferentiated mESC clones (three P1-CTCF-KO and three P2-CTCF-KO). CTCF occupancy is shown at the *Runx1* P2 promoter and a positive control gene promoter that also binds CTCF. Binding at *Runx1* P1 could not be assessed as binding is too low in undifferentiated mESCs.

Supplementary Figure 8.

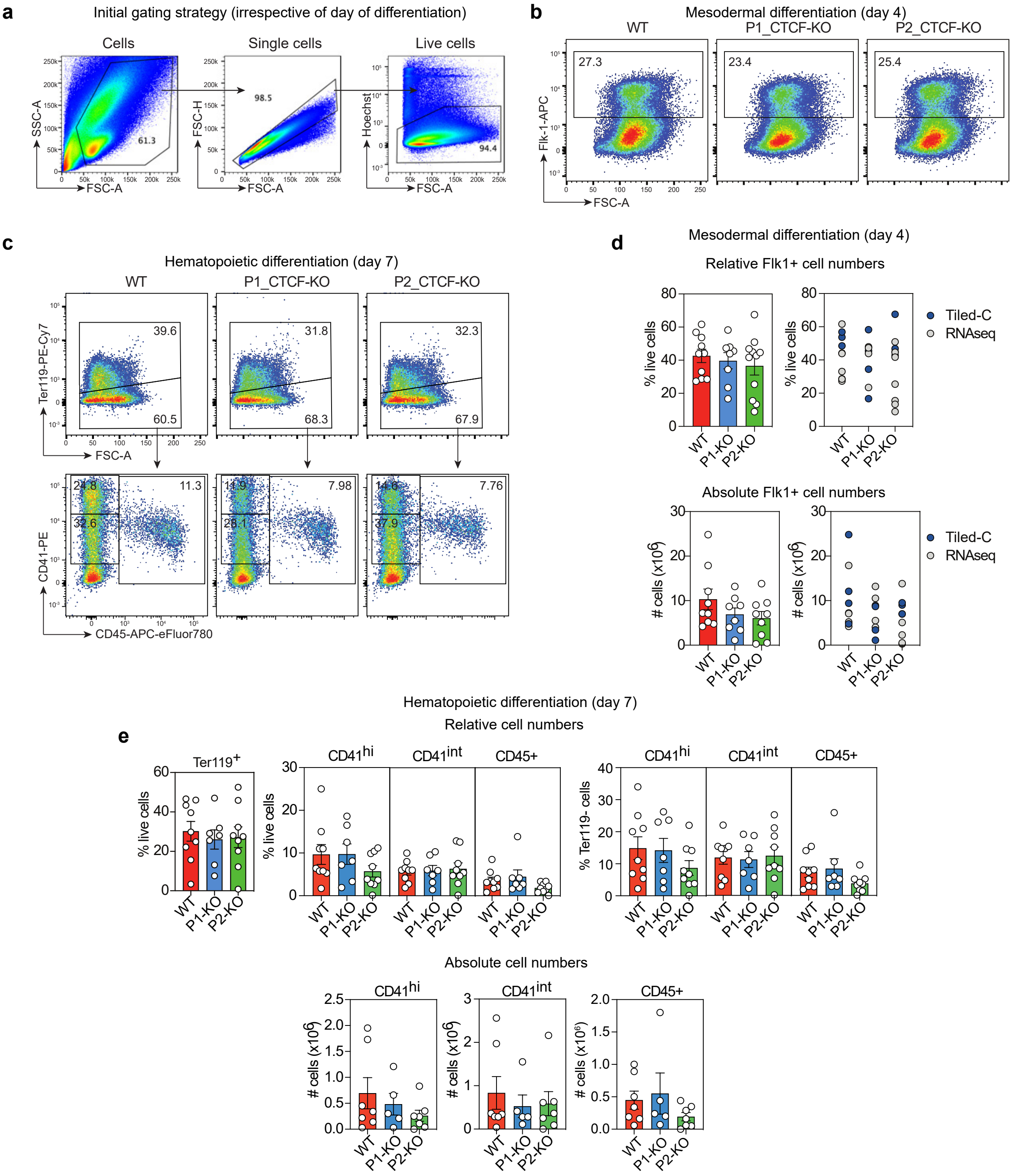

Supplemetary Figure 8 – CTCF binding upstream of *Runx1* P1 or P2 promoter is not required for hematopoietic differentiation of mESCs. a) Initial gating strategy used for all flow cytometry. Flow cytometry analysis of wild type, P1-CTCF-KO and P2-CTCF-KO mesoderm cells at differentiation day 4 (Flk1) (b) and day 7 (Ter119, CD41 and CD45) (c). Relative cell numbers (percentages of live cells or Terr119- cells) and absolute cell numbers are shown for each genotype in mesoderm (d) and hematopoietic cells (e). Results are from three independent clones per genotype and 10 independent experiments. Mesodermal differentiation wild type n=10, P1-CTCF-KO n=8, P2-CTCF-KO n=11. Hematopoietic differentiation (relative cell numbers) wild type n=9, P1-CTCF-KO n=7, P2-CTCF-KO n=9. Hematopoietic differentiation (absolute cell numbers) wild type n=7, P1-CTCF-KO n=5, P2-CTCF-KO n=7. Erythroid cells are contained within the Ter119+ gate, hematopoietic progenitors are CD41int, and megakaryocytes are contained within the CD41hi gate. Bars indicate mean and error bars indicate standard error of the mean.

Supplementary Figure 9.

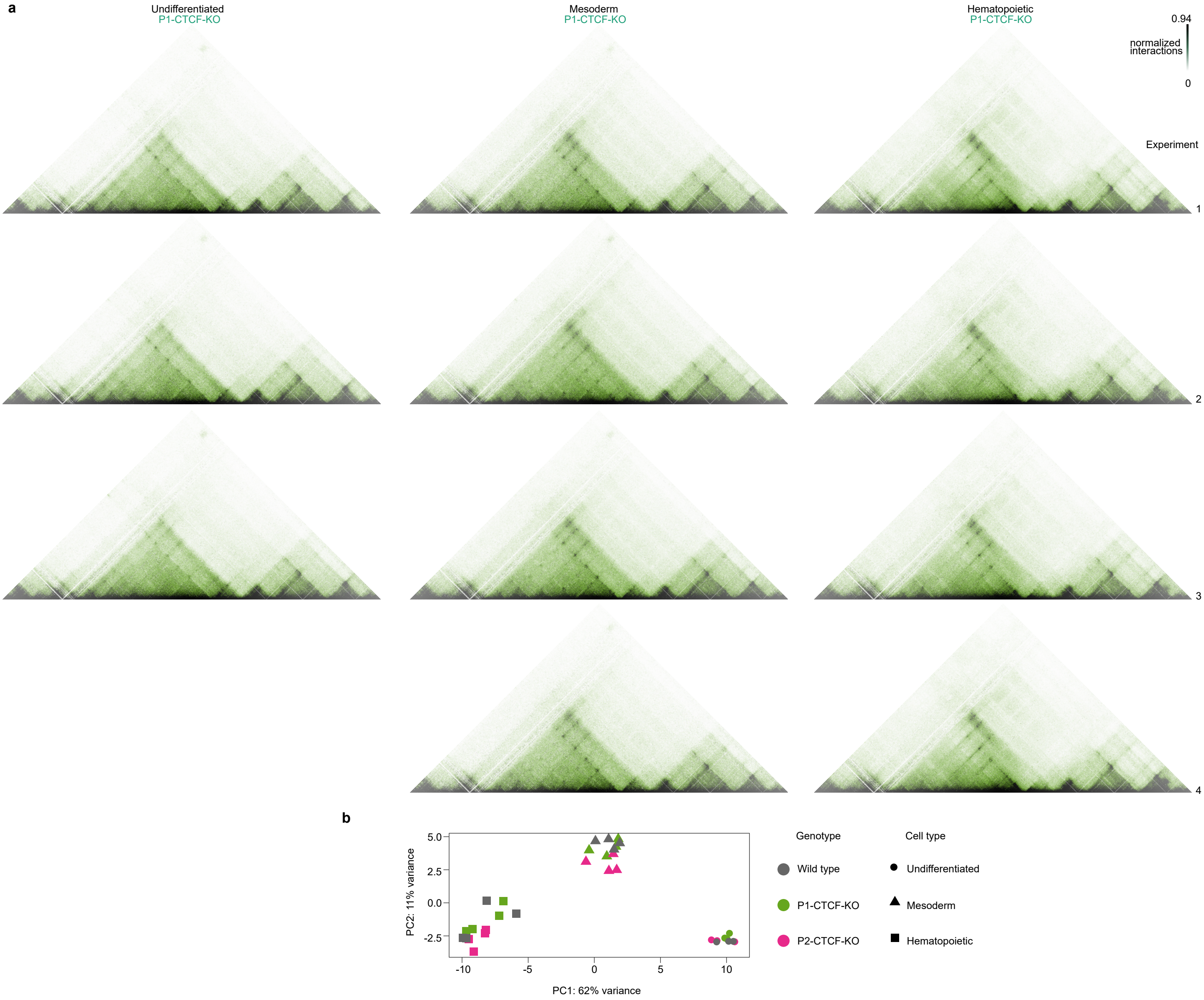

Supplemetary Figure 9 – Tiled-C matrices of individual replicates in P1-CTCF-KO cells over hematopoietic differentiation. a) Individual matrices are shown at 2 kb resolution and are total count and ICE normalized. All matrices are visualized with a threshold set at the 94th percentile. **b**) Principal component analysis (PCA) of Tiled-C data for individual replicates, with each cell type shown in a different shape and each genotype colored differently.

Supplementary Figure 10.

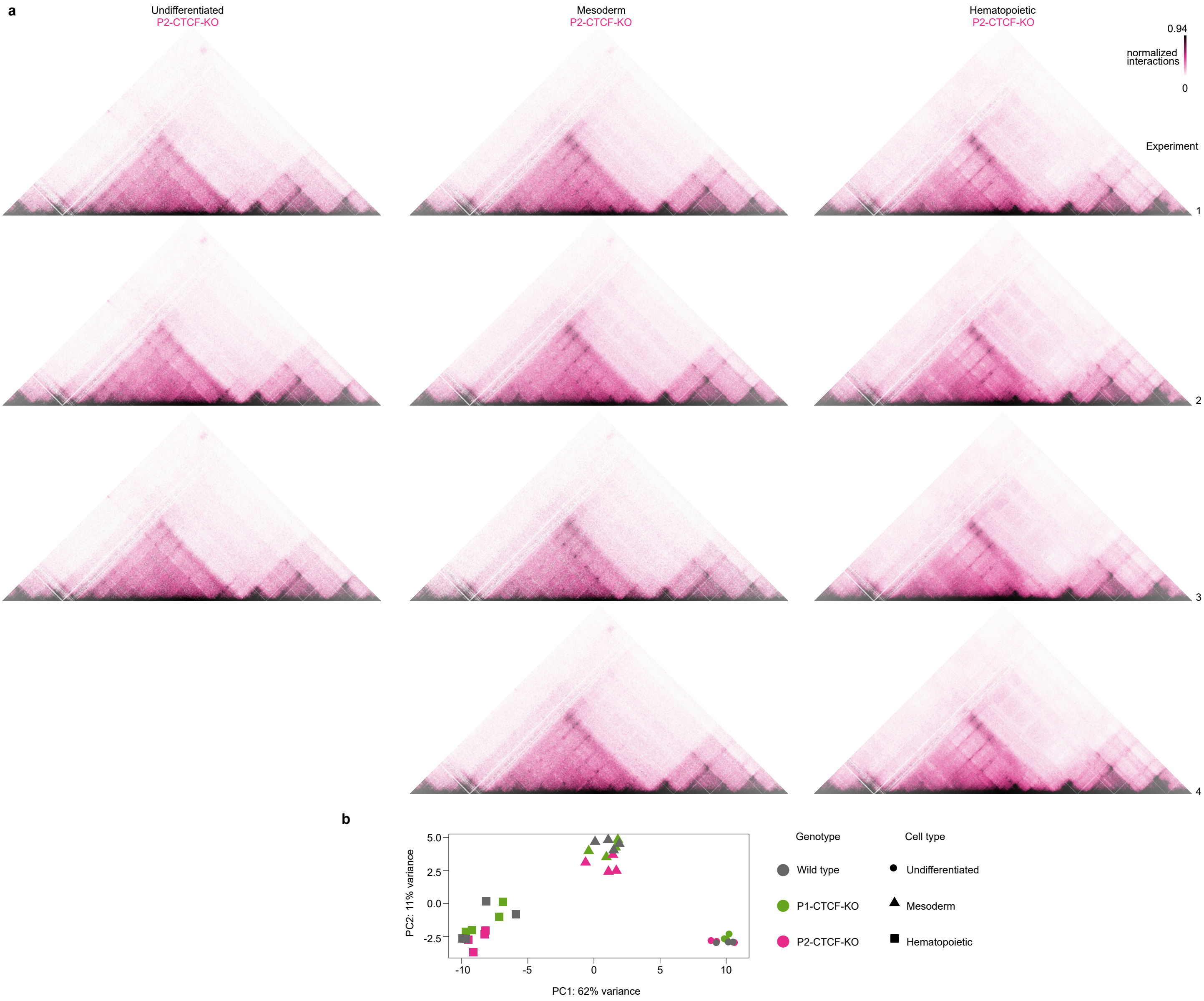

Supplemetary Figure 10 – Tiled-C matrices of individual replicates in P2-CTCF-KO cells over hematopoietic differentiation. a) Individual matrices are shown at 2 kb resolution and are total count and ICE normalized. All matrices are visualized with a threshold set at the 94th percentile. **b**) Principal component analysis (PCA) of Tiled-C data for individual replicates, with each cell type shown in a different shape and each genotype colored differently.

Supplementary Figure 11.

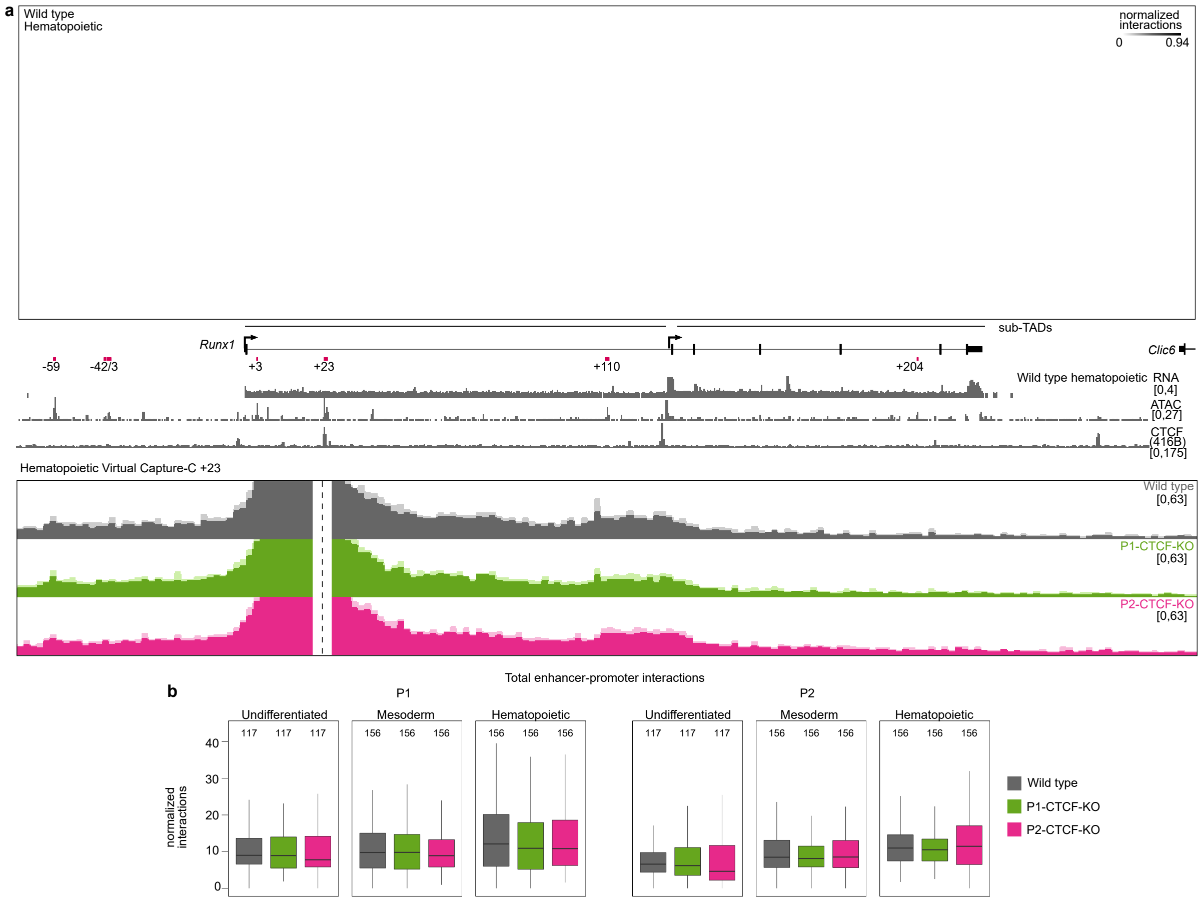

Supplemetary Figure 11 – Enhancer-promoter contacts are maintained across hematopoietic differentiation despite loss of CTCF binding and disrupted cis-interactions. a) Tiled-C matrix from wild type hematopoietic cells (2 kb resolution, threshold at the 94th percentile, n=4). *Runx1* promoters, previously published enhancers, and location of *Runx1* sub-TADs are labelled below the matrix. RPKM-normalized ATAC-seq track is shown. CPM-normalized poly(A)-minus RNA-seq (n=4) is shown. Previously published enhancer regions are indicated, numbered according to their distance from the *Runx1* start codon in exon 1. CTCF occupancy in 416B hematopoietic progenitor cells is shown. Virtual Capture-C profiles (obtained from Tiled-C data, see methods) from the viewpoint of +23 enhancer is shown in hematopoietic cells from wild type (grey track), P1-CTCF-KO (green track), and P2-CTCF-KO (pink track). Capture viewpoint over +23 enhancer is indicated by a vertical dashed line. Dark colors represent the mean reporter counts in 2 kb bins (n=4 biologically independent experiments) normalized to the total cis-interactions in each sample. Standard deviation is shown in the lighter color. **b**) Enhancer-promoter contacts quantified between *Runx1* P1 or P2 promoters and all previously published hematopoietic enhancers (Supplementary Table 2). There were no significant differences between genotypes for either promoter in any of the cell types examined (Kruskal-Wallis and Dunn's test, adjusted two-sided p = 1.0). Boxplot centre shows median, bounds of the box indicate 25th and 75th percentiles, and maxima and minima show the largest point above or below 1.5 * interquartile range. Outlying points are not shown. Data were analyzed from the total number of bins indicated above each boxplot from four biologically independent experiments.

Supplementary Figure 12.

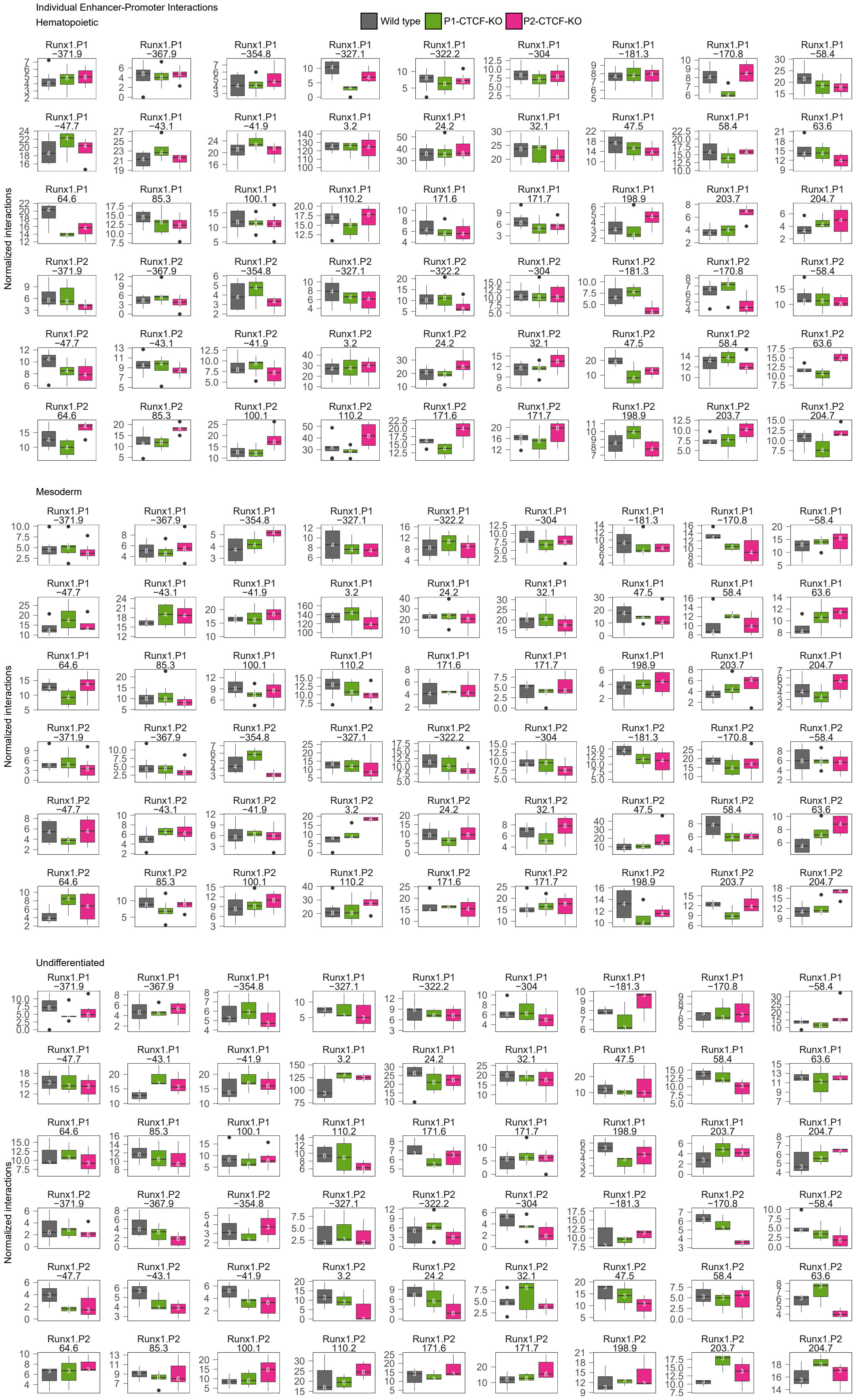

Supplemetary Figure 12 – Individual enhancer-promoter interactions are maintained despite perturbed chromatin architecture. *Legend continued on next page.*

Supplemetary Figure 12 – *Legend continued from previous page.* Enhancer-promoter contacts individually quantified between *Runx1* P1 or P2 promoters and all previously published hematopoietic enhancers (Supplementary Table 2) throughout differentiation in the cell types indicated. There were no significant differences between genotypes for any of the enhancer-promoter interactions in any of the cell types examined (Kruskal-Wallis and Dunn's test, adjusted two-sided $p = 1.0$). Boxplot centre shows median, bounds of the box indicate 25th and 75th percentiles, and maxima and minima show the largest point above or below 1.5 * interquartile range. Data were analyzed from the total number of bins indicated on each boxplot from three (undifferentiated) or four (mesoderm and hematopoietic) biologically independent experiments.

Supplementary Figure 13.

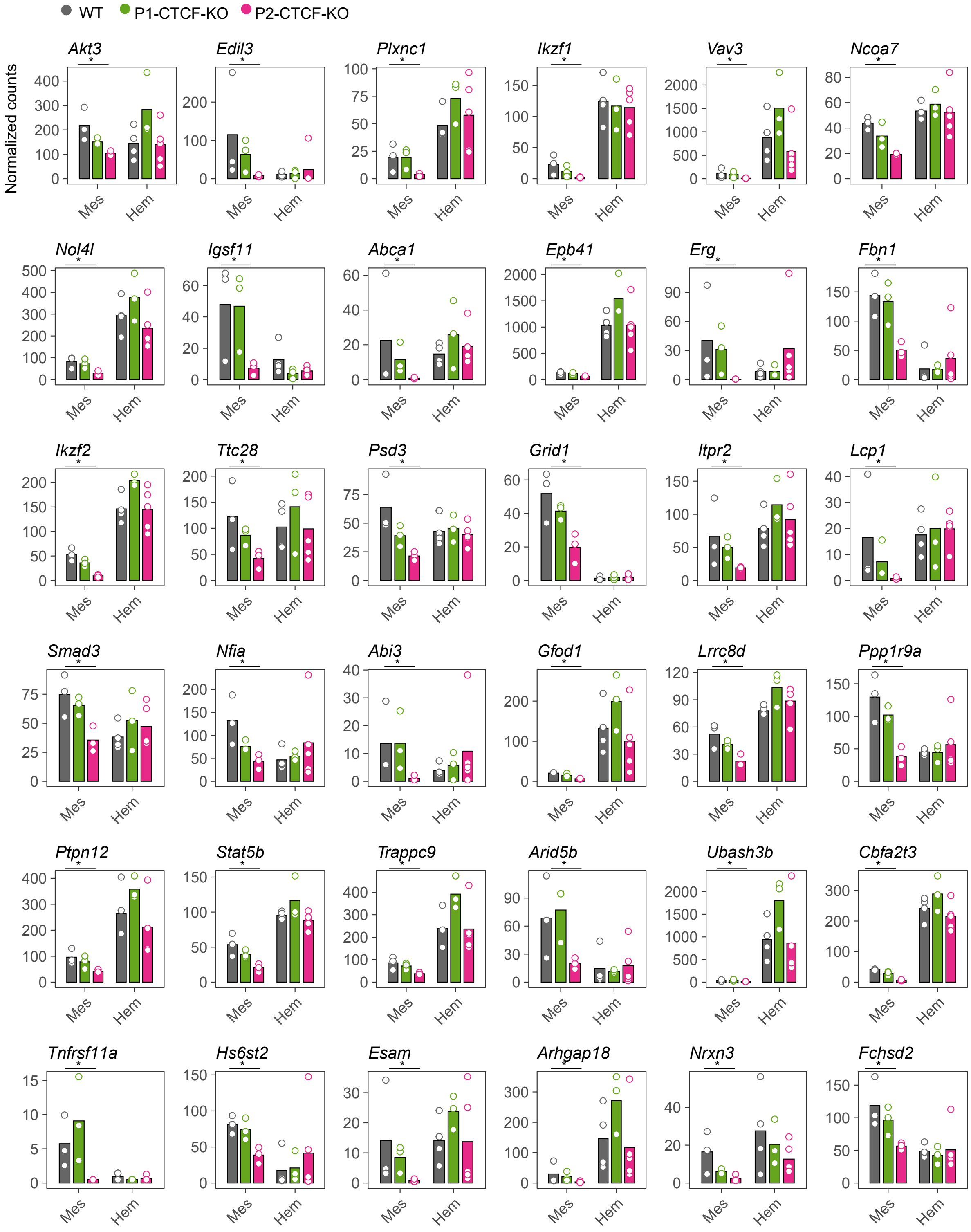

Supplemetary Figure 13 – Predicted Runx1 target genes that were downregulated in P2-CTCF-KO mesoderm. Genes predicted to be RUNX1 target genes in a KMT2A-AFF1 leukemia gene regulatory network[6] that were downregulated in P2-CTCF-KO ES-derived Flk1+ mesoderm. DEseq2, fold change>1, two-sided p-values (*): *Akt3* (.014), *Edil3* (.019), *Plxnc1* (.046), *Ikzf1* (.016), *Vav3* (.030), *Ncoa7* (.048), *Nol4l* (.015), *Igsf11* (.011), *Abca1* (.014), *Epb41* (.011), *Erg* (.0079), *Fbn1* (.0019), *Ikzf2* (1.1x10-5), *Ttc28* (.038), *Psd3* (.0079), *Grid1* (.033), *Itpr2* (.037), *Lcp1* (.028), *Smad3* (.044), *Nfia* (.0048), *Abi3* (.0046), *Gfod1* (.0045), *Lrrc8d* (.037), *Ppp1r9a* (7.5x10^{-5}) *Ptpn12*, (.038), *Stat5b* (.021), *Trappc9* (.026), *Arid5b* (.037), *Ubash3b* (.041), *Cbfa2t3* (1.1x10^{-5}), *Tnfrsf11a* (.048), *Hs6st2* (.038), *Esam* (.031), *Arhgap18* (.045), *Nrxn3* (.038), *Fchsd2* (.040). Normalized counts for each gene are shown for each genotype in mesoderm (Mes) and hematopoietic (Hem) cells. Boxplot centre shows median, bounds of the box indicate 25th and 75th percentiles, and maxima and minima show the largest point above or below 1.5 * interquartile range. N=3 biologically independent experiments (wild type, P1-CTCF-KO, P2-CTCF-KO mesoderm, P1-CTCF-KO hematopoietic), n=4 biologically independent experiments (wild type hematopoietic), n=5 biologically independent experiments (P2-CTCF-KO hematopoietic).

Supplemetary Table 1 – List of open chromatin sites in the *Runx1* TAD

mm9	start	stop	Undiff	Meso	HPC	distance to Runx1 ATG	Known element overlap	reference
chr16	92498792	92498856		Y		327.1		
chr16	92498976	92499057	Y			326.9		
chr16	92515194	92515332	Y			310.6		
chr16	92520183	92520297	Y	Y		305.6		
chr16	92544652	92544733	Y			281.2		
chr16	92566069	92566274	Y	Y		259.7		
chr16	92566562	92566632		Y		259.3		
chr16	92571567	92571768	Y			254.2		
chr16	92605770	92605858	Y			220.1		
chr16	92606055	92606271	Y			219.7		
chr16	92621095	92621162			Y	204.8	+204	[3]
chr16	92632338	92632592		Y		193.4		
chr16	92639468	92639526	Y			186.4		
chr16	92652205	92652314		Y		173.6		
chr16	92680959	92681017	Y			144.9		
chr16	92686184	92686312	Y			139.6		
chr16	92697559	92697773	Y	Y	Y	128.2	P2	[7]
chr16	92698058	92698142		Y		127.8	P2	[7]
chr16	92698987	92699142	Y	Y		126.8		
chr16	92709049	92709132	Y			116.8		
chr16	92715843	92716073		Y	Y	109.9	+110	[3]
chr16	92753499	92753649		Y		72.3		
chr16	92778300	92778757	Y	Y		47.4	+48	[8]
chr16	92778949	92779007	Y			46.9		
chr16	92785249	92785421		Y		40.6		
chr16	92787368	92787515			Y	38.4		
chr16	92801791	92802205		Y	Y	23.9	+23	[9]
chr16	92819293	92819368			Y	6.6		
chr16	92822529	92822841		Y	Y	3.2	+3	[3]
chr16	92828323	92828494		Y	Y	-2.5		
chr16	92828609	92828698		Y		-2.8		
chr16	92857265	92857329		Y		-31.4		
chr16	92867588	92867655			Y	-41.7	-42	[3]
chr16	92874454	92874521			Y	-48.6		
chr16	92884073	92884489		Y	Y	-58.4	-59	[3]
chr16	92892091	92892223		Y		-66.3		
chr16	92996475	92996601		Y		-170.6	-171	[10]
chr16	92997104	92997446		Y		-171.4	-171	[10]
chr16	92999470	92999537	Y			-173.6		
chr16	93006972	93007130	Y	Y		-181.2	-181	[10]
chr16	93020651	93020717	Y			-194.8		
chr16	93041725	93041882	Y			-215.9		
chr16	93047887	93048252	Y			-222.2		
chr16	93071978	93072322		Y		-246.3		
chr16	93103389	93103447	Y			-277.5		
chr16	93129851	93130048		Y		-304.1	-303	[11]
chr16	93137686	93137744	Y			-311.8		
chr16	93137785	93137891		Y		-311.9		
chr16	93147823	93148052		Y		-322	-321/322	[3]
chr16	93153415	93153690		Y		-327.7	-327/328	[3]
chr16	93159260	93159320	Y			-333.4		
chr16	93217970	93218034		Y		-392.1		
chr16	93293307	93293539	Y	Y	Y	-467.5		
chr16	93293634	93293701	Y			-467.8		
chr16	93329022	93329142		Y		-503.2		
chr16	93335261	93335343		Y		-509.4		

chr16	93336087	93336202		Y		-510.3
chr16	93346464	93346528		Y		-520.6
chr16	93366653	93366773		Y		-540.8
chr16	93399533	93399597		Y		-573.7
chr16	93438025	93438095		Y		-612.2
chr16	93438201	93438272		Y		-612.3
chr16	93438470	93438613	Y			-612.7
chr16	93444163	93444230	Y			-618.3
chr16	93454640	93454730	Y			-628.8
chr16	93459127	93459196	Y			-633.3
chr16	93459610	93459681	Y			-633.8
chr16	93463746	93463906	Y			-637.9
chr16	93491758	93491841	Y			-665.9
chr16	93511947	93512012	Y			-686.1
chr16	93512384	93512457	Y			-686.5
chr16	93540122	93540213		Y		-714.3
chr16	93544040	93544234	Y	Y		-718.2
chr16	93549667	93549763	Y			-723.8
chr16	93549805	93549946	Y			-724
chr16	93550018	93550202	Y			-724.2
chr16	93576908	93576984	Y			-751.1
chr16	93577055	93577199	Y			-751.2
chr16	93586814	93587022	Y			-761
chr16	93588107	93588171		Y		-762.2
chr16	93591267	93591381	Y			-765.4
chr16	93598152	93598255	Y	Y	Y	-772.3
chr16	93603965	93604398	Y	Y	Y	-778.3
chr16	93607994	93608092	Y			-782.2
chr16	93615876	93616012	Y	Y		-790.1

Supplemetary Table 2 – List of previously published *Runx1* enhancers

Distance to *Runx1* ATG (in kb)	mm9 chr	start	stop	Reference
-371	chr16	93197216	93198301	[11]
-368	chr16	93193448	93194216	[11]
-354	chr16	93180228	93181138	[11]
-327/328	chr16	93152333	93153713	[3;11]
-321/322	chr16	93147786	93148307	[3;11]
-303	chr16	93129687	93130187	[11]
-181	chr16	93006325	93007986	[10]
-171	chr16	92996191	92997200	[10]
-59	chr16	92883870	92884686	[3;11]
-48	chr16	92873240	92873850	[11]
-43	chr16	92868720	92869213	[3]
-42	chr16	92867258	92868232	[3]
+3	chr16	92822418	92822899	[3]
+23	chr16	92801742	92802272	[9;12;13;3;11]
+24	chr16	92801109	92801727	[9;3]
+32	chr16	92792657	92794853	[13]
+48	chr16	92778116	92778592	[8]
+59	chr16	92767120	92767941	[13]
+64	chr16	92760779	92761825	[13]
+87	chr16	92739637	92741585	[13]
+99	chr16	92725390	92726237	[13]
+110	chr16	92715159	92716303	[3;11]
+171	chr16	92653987	92654487	[14;15]
+199	chr16	92626889	92627053	[16]
+204	chr16	92620882	92621464	[3]

Supplemetary Table 3 – List of antibodies

Target	Fluorochrome	Manufacturer	Catalog #	Purpose	Dilution
Flk1	APC	eBioscience	17-5821-81	FACS	1:100
CD41	PE	BD Pharmigen	558040	FACS	1:400
CD45	APC-eFluor780	eBioscience	47-0451-82	FACS	1:200
Ter119	PE-Cy7	BD Pharmigen	557853	FACS	1:200
VE-cadherin	APC	eBioscience	17-1441-82	FACS	1:200
CD41	unconjugated	BD Pharmigen	553847	ICC	1:100
Runx1/2/3	unconjugated	Abcam	ab92336	ICC	1:100
CD31	unconjugated	R&D	AF3628	ICC	1:100
anti-Rat	Alexa Fluor 555	Invitrogen	A-21434	ICC	1:400
anti-Goat	Alexa Fluor 647	Invitrogen	A-21447	ICC	1:400
anti-Rabbit	Alexa Fluor 647	Invitrogen	A-11008	ICC	1:400
CTCF	unconjugated	EMD Millipore	07-729	ChIP	1:200

Supplemetary Table 4 – List of single guide RNAs

Target site	5’-3’ sequence
P2-CTCF sgRNA1	GACTGATCCTCGCGCCGTCG
P2-CTCF sgRNA2	AGCCCCGACATGACCGTGAA
P1-CTCF sgRNA1	GGGTCTCCATAGGGCAAGGC
P1-CTCF sgRNA2	GAGTCCTGTGATGATAGTCA

Supplemetary Table 5 – List of primers

Target site	5'-3' sequence	Orientation	Purpose
P2-CTCF	CCTTACTTCTCTTGGGCCTTG	F	500 bp genotyping PCR
P2-CTCF	CTGGTGGCCACTTCCTAATG	R	500 bp genotyping PCR
P2-CTCF	GAAGTGGCACCGAGTCATTTA	F	1.8 kb genotyping PCR
P2-CTCF	CCTGATCGAGCTTCGAACTAAC	R	1.8 kb genotyping PCR
P2-CTCF	CTCGTTTGCATAGAGGAGAC	F	3 kb genotyping PCR
P2-CTCF	CAGTTAGCCAGTCACGTAAG	R	3 kb genotyping PCR
P2-CTCF	GCTACTAATGTATGTGCTCGT	F	5 kb genotyping PCR
P2-CTCF	GCTCATGGTGTGTTAGAGTC	R	5 kb genotyping PCR
P2-CTCF	CGAAACAGGAATCGAGAGAC	F	ddPCR 5'1kb
P2-CTCF	GGCAGCTTTGTGTCCAG	R	ddPCR 5'1kb
P2-CTCF	CCCTCCACTTTCATCTGTG	F	ddPCR 5'100bp
P2-CTCF	CCACAGCTTCTTCCTCTTC	R	ddPCR 5'100bp
P2-CTCF	GTTAGGCGTCCTGGGAA	F	ddPCR LOA
P2-CTCF	CACAAGATCGACCCTAAGGA	R	ddPCR LOA
P2-CTCF	GGGACAGACATTAGGAAGTG	F	ddPCR 3'100bp
P2-CTCF	CTACCACCGGTCTGAGAG	R	ddPCR 3'100bp
P2-CTCF	CACTTGACACGCACTTGAAA	F	ddPCR 3'1kb
P2-CTCF	CCCTCGGTAGAGTCCCA	R	ddPCR 3'1kb
P1-CTCF	ACTTAAGTGTCCACTCCGATTA	F	500 bp genotyping PCR
P1-CTCF	GGGATTAAGCACTTCTTTAGGC	R	500 bp genotyping PCR
P1-CTCF	CCACCTATTGACCTCTTCGTTC	F	1.8 kb genotyping PCR
P1-CTCF	TGCTACTGACTAATTTGAGGGTATT	R	1.8 kb genotyping PCR
P1-CTCF	CGAGCTCCACTCAAAGAAAT	F	3 kb genotyping PCR
P1-CTCF	TCTAGGAAGGTCATGGAAATAAG	R	3 kb genotyping PCR
P1-CTCF	TACTCACCTCTCATGAAGCA	F	5 kb genotyping PCR
P1-CTCF	CCTTCTGCACAGAATGTCAA	R	5 kb genotyping PCR
P1-CTCF	GCATGGACATCTCTTGGTAA	F	ddPCR 5'1kb
P1-CTCF	CTCCTTTGCTCTCCACAAA	R	ddPCR 5'1kb
P1-CTCF	CCTGTAGTCATTTCACTTCAGAAA	F	ddPCR 5'100bp
P1-CTCF	GGGCATGAGGACGCTTA	R	ddPCR 5'100bp
P1-CTCF	GTCACCTCTGGGTCCTG	F	ddPCR LOA
P1-CTCF	GGACTTCCATGACTATCATCAC	R	ddPCR LOA
P1-CTCF	CCAAGGAAGCAGCAGTTAAA	F	ddPCR 3'100bp
P1-CTCF	CAGCCACTGAATCTCTCCTA	R	ddPCR 3'100bp
P1-CTCF	GCTTTGTGGACTGAACAGA	F	ddPCR 3'1kb
P1-CTCF	CGACTCTAAGTGTCCAGAGA	R	ddPCR 3'1kb
chr4_internal_control	GCAGGCTTGAGTAAGAAGAAG	F	ddPCR internal control
chr4_internal_control	ATTTCCCTCTGAGTCTCCTG	R	ddPCR internal control
chr1_diploid_control	GGTAGTATCTCCACCGATGA	F	ddPCR diploid control
chr1_diploid_control	CTATACTGAAGCGCATGGAC	R	ddPCR diploid control
chr6_diploid_control	AGTAGCTAGCCGTTGGTAATAG	F	ddPCR diploid control
chr6_diploid_control	GCACTGTGAGGGAAACAAAC	R	ddPCR diploid control
chr7_diploid_control	TATAGGTGCAACCCGGAAT	F	ddPCR diploid control
chr7_diploid_control	TCGGCCGACTAGTCTTTAG	R	ddPCR diploid control
CTCF_positive_control_1	GGCCAAGATAGAGATGGGTTG	F	ChIP qPCR
CTCF_positive_control_1	GTGCCTGATGCCACCTATAC	R	ChIP qPCR
CTCF_positive_control_2	CAATTACCAACTCCGTTCCT	F	ChIP qPCR
CTCF_positive_control_2	ATTGGTAGAACGACTTTCCG	R	ChIP qPCR
ChIP_negative_control_1	AAGGCTGAAATGCGGATAAA	F	ChIP qPCR
ChIP_negative_control_1	CCACTTTCCAGCTCTAGGTA	R	ChIP qPCR
ChIP_negative_control_2	GGAATGATCACGTCCTTAGC	F	ChIP qPCR
ChIP_negative_control_2	GAGATGTGTTGGGTCTGTAAA	R	ChIP qPCR
DpnII_cut_site	GTGTCACCAAAACCAGCTCA	F	3C library digestion efficiency qPCR
DpnII_cut_site	CCTGGAATCCTTTGGCTCAAG	R	3C library digestion efficiency qPCR
DpnII_cut_site	GGGCAGCTAAGATGCAAGTC	Probe	3C library digestion efficiency qPCR
Uncut_genomic_control	TGGAGGGCATATAAGTGCTACTTG	F	3C library digestion efficiency qPCR
Uncut_genomic_control	TGCTTTTGTCTTCCCCAGAGA	R	3C library digestion efficiency qPCR
Uncut_genomic_control	TGCAGGTCCAAGACACTTCTGATTCTGACA	Probe	3C library digestion efficiency qPCR

Supplementary References

[1] J. Vierstra, E. Rynes, R. Sandstrom, M. Zhang, T. Canfield, R. S. Hansen, S. Stehling-Sun, P. J. Sabo, R. Byron, R. Humbert, R. E. Thurman, A. K. Johnson, S. Vong, K. Lee, D. Bates, F. Neri, M. Diegel, E. Giste, E. Haugen, D. Dunn, M. S. Wilken, S. Josefowicz, R. Samstein, K. H. Chang, E. E. Eichler, M. De Bruijn, T. A. Reh, A. Skoultchi, A. Rudensky, S. H. Orkin, T. Papayannopoulou, P. M. Treuting, L. Selleri, R. Kaul, M. Groudine, M. A. Bender, and J. A. Stamatoyannopoulos. Mouse regulatory DNA landscapes reveal global principles of cis-regulatory evolution. *Science*, 346(6212):1007–1012, Nov 2014.

[2] L. Handoko, H. Xu, G. Li, C. Y. Ngan, E. Chew, M. Schnapp, C. W. Lee, C. Ye, J. L. Ping, F. Mulawadi, E. Wong, J. Sheng, Y. Zhang, T. Poh, C. S. Chan, G. Kunarso, A. Shahab, G. Bourque, V. Cacheux-Rataboul, W. K. Sung, Y. Ruan, and C. L. Wei. CTCF-mediated functional chromatin interactome in pluripotent cells. *Nat. Genet.*, 43(7):630–638, Jun 2011.

[3] J. Schutte, H. Wang, S. Antoniou, A. Jarratt, N. K. Wilson, J. Riepsaame, F. J. Calero-Nieto, V. Moignard, S. Basilico, S. J. Kinston, R. L. Hannah, M. C. Chan, S. T. Nürnberg, W. H. Ouwehand, N. Bonzanni, M. F. de Bruijn, and B. Göttgens. An experimentally validated network of nine haematopoietic transcription factors reveals mechanisms of cell state stability. *Elife*, 5:e11469, Feb 2016.

[4] A. Siepel, G. Bejerano, J. S. Pedersen, A. S. Hinrichs, M. Hou, K. Rosenbloom, H. Clawson, J. Spieth, L. W. Hillier, S. Richards, G. M. Weinstock, R. K. Wilson, R. A. Gibbs, W. J. Kent, W. Miller, and D. Haussler. Evolutionarily conserved elements in vertebrate, insect, worm, and yeast genomes. *Genome Res.*, 15(8): 1034–1050, Aug 2005.

[5] W. J. Kent, C. W. Sugnet, T. S. Furey, K. M. Roskin, T. H. Pringle, A. M. Zahler, and D. Haussler. The human genome browser at UCSC. *Genome Res*, 12(6):996–1006, Jun 2002.

[6] J. R. Harman, R. Thorne, M. Jamilly, M. Tapia, N. T. Crump, S. Rice, R. Beveridge, E. Morrissey, M. F. T. R. de Bruijn, I. Roberts, A. Roy, T. A. Fulga, and T. A. Milne. A KMT2A-AFF1 gene regulatory network highlights the role of core transcription factors and reveals the regulatory logic of key downstream target genes. *Genome Res*, Jun 2021.

[7] D. Levanon and Y. Groner. Structure and regulated expression of mammalian RUNX genes. *Oncogene*, 23 (24):4211–4219, May 2004.

[8] C. K. Cheng, T. H. Y. Wong, T. S. K. Wan, A. Z. Wang, N. P. H. Chan, N. C. N. Chan, C. K. Li, and M. H. L. Ng. RUNX1 upregulation via disruption of long-range transcriptional control by a novel t(5;21)(q13;q22) translocation in acute myeloid leukemia. *Mol Cancer*, 17(1):133, 08 2018.

[9] W. T. Nottingham, A. Jarratt, M. Burgess, C. L. Speck, J. F. Cheng, S. Prabhakar, E. M. Rubin, P. S. Li, J. Sloane-Stanley, J. Kong-A-San, and M. F. de Bruijn. Runx1-mediated hematopoietic stem-cell emergence is controlled by a Gata/Ets/SCL-regulated enhancer. *Blood*, 110(13):4188–4197, Dec 2007.

[10] L. T. G. Harland, C. S. Simon, A. D. Senft, I. Costello, L. Greder, I. Imaz-Rosshandler, B. Göttgens, J. C. Marioni, E. K. Bikoff, C. Porcher, M. F. T. R. de Bruijn, and E. J. Robertson. The T-box transcription factor Eomesodermin governs haemogenic competence of yolk sac mesodermal progenitors. *Nat Cell Biol*, 23(1): 61–74, 01 2021.

[11] J. Marsman, A. Thomas, M. Osato, J. M. O'Sullivan, and J. A. Horsfield. A DNA Contact Map for the Mouse Runx1 Gene Identifies Novel Haematopoietic Enhancers. *Sci Rep*, 7(1):13347, 10 2017.

[12] T. Bee, G. Swiers, S. Muroi, A. Pozner, W. Nottingham, A. C. Santos, P. S. Li, I. Taniuchi, and M. F. de Bruijn. Nonredundant roles for Runx1 alternative promoters reflect their activity at discrete stages of developmental hematopoiesis. *Blood*, 115(15):3042–3050, Apr 2010.

[13] C. E. Ng, T. Yokomizo, N. Yamashita, B. Cirovic, H. Jin, Z. Wen, Y. Ito, and M. Osato. A Runx1 intronic enhancer marks hemogenic endothelial cells and hematopoietic stem cells. *Stem Cells*, 28(10):1869–1881, Oct 2010.

[14] P. Cauchy, S. R. James, J. Zacarias-Cabeza, A. Ptasinska, M. R. Imperato, S. A. Assi, J. Piper, M. Canestraro, M. Hoogenkamp, M. Raghavan, J. Loke, S. Akiki, S. J. Clokie, S. J. Richards, D. R. Westhead, M. J.

Griffiths, S. Ott, C. Bonifer, and P. N. Cockerill. Chronic FLT3-ITD Signaling in Acute Myeloid Leukemia Is Connected to a Specific Chromatin Signature. *Cell Rep*, 12(5):821–836, Aug 2015.

[15] S. R. Fitch, C. Kapeni, A. Tsitsopoulou, N. K. Wilson, B. Göttgens, M. F. de Bruijn, and K. Ottersbach. Gata3 targets Runx1 in the embryonic haematopoietic stem cell niche. *IUBMB Life*, 72(1):45–52, 01 2020.

[16] K. Ortt, E. Raveh, U. Gat, and S. Sinha. A chromatin immunoprecipitation screen in mouse keratinocytes reveals Runx1 as a direct transcriptional target of DeltaNp63. *J Cell Biochem*, 104(4):1204–1219, Jul 2008.
