# Supplementary material for: Dynamic Runx1 chromatin boundaries affect gene expression in hematopoietic development
Source: Nat Commun. 2022 Feb 9;13:773. doi: 10.1038/s41467-022-28376-8 (PMC8828719; doi:10.1038/s41467-022-28376-8)
Supplement: Supplementary file 3 — Reporting Summary [file 41467_2022_28376_MOESM3_ESM.pdf]

nature portfolio

Corresponding author(s): Marella de Bruijn

Last updated by author(s): Dec 15, 2021

Reporting Summary

Nature Portfolio wishes to improve the reproducibility of the work that we publish. This form provides structure for consistency and transparency in reporting. For further information on Nature Portfolio policies, see our Editorial Policies and the Editorial Policy Checklist.

Statistics

For all statistical analyses, confirm that the following items are present in the figure legend, table legend, main text, or Methods section.

n/a	Confirmed	
☐	☒	The exact sample size (*n*) for each experimental group/condition, given as a discrete number and unit of measurement
☐	☒	A statement on whether measurements were taken from distinct samples or whether the same sample was measured repeatedly
☐	☒	The statistical test(s) used AND whether they are one- or two-sided<br>*Only common tests should be described solely by name; describe more complex techniques in the Methods section.*
☒	☐	A description of all covariates tested
☐	☒	A description of any assumptions or corrections, such as tests of normality and adjustment for multiple comparisons
☐	☒	A full description of the statistical parameters including central tendency (e.g. means) or other basic estimates (e.g. regression coefficient) AND variation (e.g. standard deviation) or associated estimates of uncertainty (e.g. confidence intervals)
☐	☒	For null hypothesis testing, the test statistic (e.g. *F*, *t*, *r*) with confidence intervals, effect sizes, degrees of freedom and *P* value noted<br>*Give P values as exact values whenever suitable.*
☒	☐	For Bayesian analysis, information on the choice of priors and Markov chain Monte Carlo settings
☒	☐	For hierarchical and complex designs, identification of the appropriate level for tests and full reporting of outcomes
☒	☐	Estimates of effect sizes (e.g. Cohen's *d*, Pearson's *r*), indicating how they were calculated

Our web collection on statistics for biologists contains articles on many of the points above.

Software and code

Policy information about availability of computer code

Data collection	BD FACSDiva (BD Biosciences, version 8.0.1)<br>QuantaSoft (Bio-Rad, version 1.7.4)
Data analysis	R 3.6.0<br>Python 3.7.5<br>MACS 2.0.10<br>meme 4.9.1_1<br>CCSeqBasic CM5<br>fastqc 0.10.1<br>trim_galore 0.3.1<br>bismark 0.20.0<br>STAR 2.6.1d<br>deeptools 2.2.2 and 3.0.1<br>HiCPro 2.11.1<br>DESeq2 1.30.1<br>NGSeqBasic VS2.0<br>FlowJo (TreeStar, version 10.8)<br>Custom code available of github https://github.com/d0minicO/Owens_et_al_Tiled-C

For manuscripts utilizing custom algorithms or software that are central to the research but not yet described in published literature, software must be made available to editors and reviewers. We strongly encourage code deposition in a community repository (e.g. GitHub). See the Nature Portfolio guidelines for submitting code & software for further information.

Data

Policy information about availability of data

All manuscripts must include a data availability statement. This statement should provide the following information, where applicable:

- Accession codes, unique identifiers, or web links for publicly available datasets
- A description of any restrictions on data availability
- For clinical datasets or third party data, please ensure that the statement adheres to our policy

The sequencing data generated in this study have been deposited in the GEO database under accession code GSE184490
E14 mESC CTCF ChIP-seq data used in this study are available in the GEO database under accession code GSE28247
416B H3K27ac ChIP-seq data used in this study are available in the GEO database under accession code GSE69776
416B and E14 mESC DNaseI-seq data used in this study are available in the GEO database under accession code GSE37074

Field-specific reporting

Please select the one below that is the best fit for your research. If you are not sure, read the appropriate sections before making your selection.

☒ Life sciences ☐ Behavioural & social sciences ☐ Ecological, evolutionary & environmental sciences

For a reference copy of the document with all sections, see nature.com/documents/nr-reporting-summary-flat.pdf

Life sciences study design

All studies must disclose on these points even when the disclosure is negative.

Sample size	We did not perform sample size calculation. Experiments were performed in 3 biological replicates as is common in the field (Oudelaar et al., Nature Communication 2020) and the observed biological effects of interest were very robust between replicates.
Data exclusions	No data were excluded.
Replication	At least three independent experiments were performed as described and all attempts at replication were successful.
Randomization	Randomization was not performed. Control of covariates was not relevant for our study as the different groups represent different cell populations of which multiple characteristics were analyzed.
Blinding	Blinding was not performed during data collection due to technical complexity of the experiments. Blinding of samples during bioinformatics analyses was not performed as all samples were processed using the same pipelines and scripts.

Reporting for specific materials, systems and methods

We require information from authors about some types of materials, experimental systems and methods used in many studies. Here, indicate whether each material, system or method listed is relevant to your study. If you are not sure if a list item applies to your research, read the appropriate section before selecting a response.

Materials & experimental systems

n/a	Involved in the study	
☐	☒	Antibodies
☐	☒	Eukaryotic cell lines
☒	☐	Palaeontology and archaeology
☒	☐	Animals and other organisms
☒	☐	Human research participants
☒	☐	Clinical data
☒	☐	Dual use research of concern

Methods

n/a	Involved in the study	
☐	☒	ChIP-seq
☐	☒	Flow cytometry
☒	☐	MRI-based neuroimaging

Antibodies

Antibodies used

anti-Flk1-APC (eBioscience, 17-5821-81) dilution 1:100
anti-CD41-PE (BD Pharmigen, 558040) dilution 1:400
anti-CD45-APC-eFluor780 (eBioscience, 47-0451-82) dilution 1:200
anti-Ter119-PE-Cy7 (BD Pharmigen, 557853) dilution 1:200
anti-VE-cadherin-APC (eBioscience, 17-1441-82) dilution 1:200
anti-CD41-unconjugated (BD Pharmigen, 553847) dilution 1:100
anti-Runx1/2/3-unconjugated (Abcam, ab92336) dilution 1:100

anti-CD31-unconjugated (R&D, AF3628) dilution 1:100
anti-Rat-AlexaFluor555 (Invitrogen, A-21434) dilution 1:400
anti-Goat-AlexaFluor647 (Invitrogen, A-21447) dilution 1:400
anti-Rabbit-AlexaFluor647 (Invitrogen, A-11008) dilution 1:400
anti-CTCF-unconjugated (EMD Millipore, 07-729) dilution 1:200

Validation

anti-Flk1-APC (eBioscience, 17-5821-81) has been validated for flow cytometry on bEnd-3 cell line by the manufacturer.
anti-CD41-PE (BD Pharmigen, 558040) has been validated for flow cytometry on mouse platelets by the manufacturer.
anti-CD45-APC-eFluor780 (eBioscience, 47-0451-82) has been validated for immunohistochemistry, immunocytochemistry and flow cytometry on C57BL/6 bone marrow cells by the manufacturer.
anti-Ter119-PE-Cy7 (BD Pharmigen, 557853) has been validated for flow cytometry by the manufacturer.
anti-VE-cadherin-APC (eBioscience, 17-1441-82) has been validated for flow cytometry on bEnd-3 cell line by the manufacturer.
anti-CD41-unconjugated (BD Pharmigen, 553847) has been validated for flow cytometry, cytotoxicity, depletion, immunohistochemistry, immunoprecipitation, induction, inhibition by the manufacturer.
anti-Runx1/2/3-unconjugated (Abcam, ab92336) has been validated for flow cytometry, western blot, immunohistochemistry and immunoprecipitation on Molt-4 cells by the manufacturer.
anti-CD31-unconjugated (R&D, AF3628) has been validated for flow cytometry, western blot, immunohistochemistry and immunoprecipitation on mouse splenocytes by the manufacturer.
anti-Rat-AlexaFluor555 (Invitrogen, A-21434) has been validated for flow cytometry, immunohistochemistry and immunocytochemistry on A549 cells stained with alpha Tubulin (YL1/2) Rat Monoclonal Antibody by the manufacturer.
anti-Goat-AlexaFluor647 (Invitrogen, A-21447) has been validated for flow cytometry, western blot, immunohistochemistry and immunocytochemistry on mouse lung tissue stained with anti-SPC Goat Monoclonal Antibody by the manufacturer.
anti-Rabbit-AlexaFluor647 (Invitrogen, A-11008) has been validated for flow cytometry, western blot, immunohistochemistry and immunocytochemistry on HeLa cells stained with alpha Tubulin Rabbit Polyclonal Antibody by the manufacturer.
anti-CTCF-unconjugated (EMD Millipore, 07-729) has been validated for chromatin immunoprecipitation (ChIP), ChIP-seq and western blot on HeLa nuclear extract by the manufacturer.

Eukaryotic cell lines

Policy information about cell lines

Cell line source(s)

Mouse embryonic stem cells (E14-TG2a) were originally obtained from the University of Edinburgh via Andrew Smith and have been maintained in our in-house facility for 3 decades. These cells are also commercially available from ATCC (CRL-1821).
416B mouse immortalized myeloid progenitor cells (Dexter et al., Nature 1979) were a kind gift of T. Enver, University College London. This cell line is not commercially available.

Authentication

E14-TG2a embryonic stem cells have been extensively authenticated previously by blastocyst injection.
416B myeloid progenitors were validated by assessing the expression of the hematopoietic progenitor marker CD34 by RT-PCR.

Mycoplasma contamination

Parental cells tested negative for mycoplasma contamination. Individual clones were not tested.

Commonly misidentified lines
(See ICLAC register)

No commonly misidentified lines were used.

ChIP-seq

Data deposition

☒ Confirm that both raw and final processed data have been deposited in a public database such as GEO.

☒ Confirm that you have deposited or provided access to graph files (e.g. BED files) for the called peaks.

Data access links
May remain private before publication.

The sequencing data generated in this study have been deposited in the GEO database under accession code GSE184490
https://www.ncbi.nlm.nih.gov/geo/query/acc.cgi?acc=GSE184490

Files in database submission

GSM5590037 Tiled-C_P1-CTCF-KO_Undiff_bioRep_E7.1_seqRun1
GSM5590038 Tiled-C_P1-CTCF-KO_Undiff_bioRep_E7.2_seqRun1
GSM5590039 Tiled-C_P1-CTCF-KO_Undiff_bioRep_E9.1_seqRun1
GSM5590040 Tiled-C_P1-CTCF-KO_Flk1_bioRep_E7.2_seqRun1
GSM5590041 Tiled-C_P1-CTCF-KO_Flk1_bioRep_E9.1_seqRun1
GSM5590042 Tiled-C_P1-CTCF-KO_Flk1_bioRep_E7.1_seqRun1
GSM5590043 Tiled-C_P1-CTCF-KO_Flk1_bioRep_E7.1-2_seqRun1
GSM5590044 Tiled-C_P1-CTCF-KO_CD41_bioRep_E7.2_seqRun1
GSM5590045 Tiled-C_P1-CTCF-KO_CD41_bioRep_E9.1_seqRun1
GSM5590046 Tiled-C_P1-CTCF-KO_CD41_bioRep_E7.1_seqRun1
GSM5590047 Tiled-C_P1-CTCF-KO_CD41_bioRep_E7.1-2_seqRun1
GSM5590048 Tiled-C_P2-CTCF-KO_Undiff_bioRep_D2.1_seqRun1
GSM5590049 Tiled-C_P2-CTCF-KO_Undiff_bioRep_D10.1_seqRun1
GSM5590050 Tiled-C_P2-CTCF-KO_Undiff_bioRep_E9.1_seqRun1
GSM5590051 Tiled-C_P2-CTCF-KO_Flk1_bioRep_D10.1_seqRun1
GSM5590052 Tiled-C_P2-CTCF-KO_Flk1_bioRep_E9.1_seqRun1
GSM5590053 Tiled-C_P2-CTCF-KO_Flk1_bioRep_D2.1_seqRun1

GSM5590054 Tiled-C_P2-CTCF-KO_Flk1_bioRep_D2.1-2_seqRun1
GSM5590055 Tiled-C_P2-CTCF-KO_CD41_bioRep_D10.1_seqRun1
GSM5590056 Tiled-C_P2-CTCF-KO_CD41_bioRep_E9.1_seqRun1
GSM5590057 Tiled-C_P2-CTCF-KO_CD41_bioRep_D2.1_seqRun1
GSM5590058 Tiled-C_P2-CTCF-KO_CD41_bioRep_D2.1-2_seqRun1
GSM5590059 Tiled-C_Wild-type_Undiff_bioRep_C6.1_seqRun1
GSM5590060 Tiled-C_Wild-type_Undiff_bioRep_C8.1_seqRun1
GSM5590061 Tiled-C_Wild-type_Undiff_bioRep_B9.3_seqRun1
GSM5590062 Tiled-C_Wild-type_Flk1_bioRep_C8.1_seqRun1
GSM5590063 Tiled-C_Wild-type_Flk1_bioRep_B9.3_seqRun1
GSM5590064 Tiled-C_Wild-type_Flk1_bioRep_C6.1_seqRun1
GSM5590065 Tiled-C_Wild-type_Flk1_bioRep_C6.1-2_seqRun1
GSM5590066 Tiled-C_Wild-type_CD41_bioRep_C8.1_seqRun1
GSM5590067 Tiled-C_Wild-type_CD41_bioRep_B9.3_seqRun1
GSM5590068 Tiled-C_Wild-type_CD41_bioRep_C6.1_seqRun1
GSM5590069 Tiled-C_Wild-type_CD41_bioRep_C6.1-2_seqRun1
GSM5590070 Tiled-C_P1-CTCF-KO_Undiff_bioRep_E7.1_seqRun2
GSM5590071 Tiled-C_P1-CTCF-KO_Undiff_bioRep_E7.2_seqRun2
GSM5590072 Tiled-C_P1-CTCF-KO_Undiff_bioRep_E9.1_seqRun2
GSM5590073 Tiled-C_P1-CTCF-KO_Flk1_bioRep_E7.2_seqRun2
GSM5590074 Tiled-C_P1-CTCF-KO_Flk1_bioRep_E9.1_seqRun2
GSM5590075 Tiled-C_P1-CTCF-KO_Flk1_bioRep_E7.1_seqRun2
GSM5590076 Tiled-C_P1-CTCF-KO_Flk1_bioRep_E7.1-2_seqRun2
GSM5590077 Tiled-C_P1-CTCF-KO_CD41_bioRep_E7.2_seqRun2
GSM5590078 Tiled-C_P1-CTCF-KO_CD41_bioRep_E9.1_seqRun2
GSM5590079 Tiled-C_P1-CTCF-KO_CD41_bioRep_E7.1_seqRun2
GSM5590080 Tiled-C_P1-CTCF-KO_CD41_bioRep_E7.1-2_seqRun2
GSM5590081 Tiled-C_P2-CTCF-KO_Undiff_bioRep_D2.1_seqRun2
GSM5590082 Tiled-C_P2-CTCF-KO_Undiff_bioRep_D10.1_seqRun2
GSM5590083 Tiled-C_P2-CTCF-KO_Undiff_bioRep_E9.1_seqRun2
GSM5590084 Tiled-C_P2-CTCF-KO_Flk1_bioRep_D10.1_seqRun2
GSM5590085 Tiled-C_P2-CTCF-KO_Flk1_bioRep_E9.1_seqRun2
GSM5590086 Tiled-C_P2-CTCF-KO_Flk1_bioRep_D2.1_seqRun2
GSM5590087 Tiled-C_P2-CTCF-KO_Flk1_bioRep_D2.1-2_seqRun2
GSM5590088 Tiled-C_P2-CTCF-KO_CD41_bioRep_D10.1_seqRun2
GSM5590089 Tiled-C_P2-CTCF-KO_CD41_bioRep_E9.1_seqRun2
GSM5590090 Tiled-C_P2-CTCF-KO_CD41_bioRep_D2.1_seqRun2
GSM5590091 Tiled-C_P2-CTCF-KO_CD41_bioRep_D2.1-2_seqRun2
GSM5590092 Tiled-C_Wild-type_Undiff_bioRep_C6.1_seqRun2
GSM5590093 Tiled-C_Wild-type_Undiff_bioRep_C8.1_seqRun2
GSM5590094 Tiled-C_Wild-type_Undiff_bioRep_B9.3_seqRun2
GSM5590095 Tiled-C_Wild-type_Flk1_bioRep_C8.1_seqRun2
GSM5590096 Tiled-C_Wild-type_Flk1_bioRep_B9.3_seqRun2
GSM5590097 Tiled-C_Wild-type_Flk1_bioRep_C6.1_seqRun2
GSM5590098 Tiled-C_Wild-type_Flk1_bioRep_C6.1-2_seqRun2
GSM5590099 Tiled-C_Wild-type_CD41_bioRep_C8.1_seqRun2
GSM5590100 Tiled-C_Wild-type_CD41_bioRep_B9.3_seqRun2
GSM5590101 Tiled-C_Wild-type_CD41_bioRep_C6.1_seqRun2
GSM5590102 Tiled-C_Wild-type_CD41_bioRep_C6.1-2_seqRun2
GSM5590103 RNA-seq-A-minus_Wild-type_416B_bioRep1_Lane1
GSM5590104 RNA-seq-A-minus_Wild-type_416B_bioRep1_Lane2
GSM5590105 RNA-seq-A-minus_Wild-type_416B_bioRep1_Lane3
GSM5590106 RNA-seq-A-minus_Wild-type_416B_bioRep1_Lane4
GSM5590107 RNA-seq-A-minus_Wild-type_416B_bioRep2_Lane1
GSM5590108 RNA-seq-A-minus_Wild-type_416B_bioRep2_Lane2
GSM5590109 RNA-seq-A-minus_Wild-type_416B_bioRep2_Lane3
GSM5590110 RNA-seq-A-minus_Wild-type_416B_bioRep2_Lane4
GSM5590111 RNA-seq-A-minus_P1-CTCF-KO_Flk1_bioRep_E7.1
GSM5590112 RNA-seq-A-minus_P1-CTCF-KO_Flk1_bioRep_E7.2
GSM5590113 RNA-seq-A-minus_P1-CTCF-KO_Flk1_bioRep_E9.1
GSM5590114 RNA-seq-A-minus_P1-CTCF-KO_CD41_bioRep_E7.1
GSM5590115 RNA-seq-A-minus_P1-CTCF-KO_CD41_bioRep_E7.2
GSM5590116 RNA-seq-A-minus_P1-CTCF-KO_CD41_bioRep_E9.1
GSM5590117 RNA-seq-A-minus_P2-CTCF-KO_Flk1_bioRep_D2.1
GSM5590118 RNA-seq-A-minus_P2-CTCF-KO_Flk1_bioRep_D10.1
GSM5590119 RNA-seq-A-minus_P2-CTCF-KO_Flk1_bioRep_E9.1
GSM5590120 RNA-seq-A-minus_P2-CTCF-KO_CD41_bioRep_D2.1
GSM5590121 RNA-seq-A-minus_P2-CTCF-KO_CD41_bioRep_D10.1
GSM5590122 RNA-seq-A-minus_P2-CTCF-KO_CD41_bioRep_D10.1-2
GSM5590123 RNA-seq-A-minus_P2-CTCF-KO_CD41_bioRep_D2.1-2
GSM5590124 RNA-seq-A-minus_P2-CTCF-KO_CD41_bioRep_E9.1
GSM5590125 RNA-seq-A-minus_Wild-type_Undiff_bioRep_E14_techRep1
GSM5590126 RNA-seq-A-minus_Wild-type_Undiff_bioRep_E14_techRep2
GSM5590127 RNA-seq-A-minus_Wild-type_Flk1_bioRep_C6.1
GSM5590128 RNA-seq-A-minus_Wild-type_Flk1_bioRep_C8.1
GSM5590129 RNA-seq-A-minus_Wild-type_Flk1_bioRep_B9.3

GSM5590130 RNA-seq-A-minus_Wild-type_CD41_bioRep_C6.1
GSM5590131 RNA-seq-A-minus_Wild-type_CD41_bioRep_C8.1
GSM5590132 RNA-seq-A-minus_Wild-type_CD41_bioRep_B9.3
GSM5590133 RNA-seq-A-minus_Wild-type_CD41_bioRep_B9.3-2
GSM5590134 Input-ChIP-seq_P1-CTCF-KO_Undiff_E7.1_seqRun1
GSM5590135 Input-ChIP-seq_P1-CTCF-KO_Undiff_E7.1_seqRun2
GSM5590136 Input-ChIP-seq_P1-CTCF-KO_Undiff_E7.2_seqRun1
GSM5590137 Input-ChIP-seq_P1-CTCF-KO_Undiff_E7.2_seqRun2
GSM5590138 Input-ChIP-seq_P1-CTCF-KO_Undiff_E9.1_seqRun1
GSM5590139 Input-ChIP-seq_P1-CTCF-KO_Undiff_E9.1_seqRun2
GSM5590140 Input-ChIP-seq_P2-CTCF-KO_Undiff_D10.1_seqRun1
GSM5590141 Input-ChIP-seq_P2-CTCF-KO_Undiff_D10.1_seqRun2
GSM5590142 Input-ChIP-seq_P2-CTCF-KO_Undiff_D2.1_seqRun1
GSM5590143 Input-ChIP-seq_P2-CTCF-KO_Undiff_D2.1_seqRun2
GSM5590144 Input-ChIP-seq_P2-CTCF-KO_Undiff_E9.1_seqRun1
GSM5590145 Input-ChIP-seq_P2-CTCF-KO_Undiff_E9.1_seqRun2
GSM5590146 CTCF-ChIP-seq_P1-CTCF-KO_Undiff_E7.1_seqRun1
GSM5590147 CTCF-ChIP-seq_P1-CTCF-KO_Undiff_E7.1_seqRun2
GSM5590148 CTCF-ChIP-seq_P1-CTCF-KO_Undiff_E7.2_seqRun1
GSM5590149 CTCF-ChIP-seq_P1-CTCF-KO_Undiff_E7.2_seqRun2
GSM5590150 CTCF-ChIP-seq_P1-CTCF-KO_Undiff_E9.1_seqRun1
GSM5590151 CTCF-ChIP-seq_P1-CTCF-KO_Undiff_E9.1_seqRun2
GSM5590152 CTCF-ChIP-seq_P2-CTCF-KO_Undiff_D10.1_seqRun1
GSM5590153 CTCF-ChIP-seq_P2-CTCF-KO_Undiff_D10.1_seqRun2
GSM5590154 CTCF-ChIP-seq_P2-CTCF-KO_Undiff_D2.1_seqRun1
GSM5590155 CTCF-ChIP-seq_P2-CTCF-KO_Undiff_D2.1_seqRun2
GSM5590156 CTCF-ChIP-seq_P2-CTCF-KO_Undiff_E9.1_seqRun1
GSM5590157 CTCF-ChIP-seq_P2-CTCF-KO_Undiff_E9.1_seqRun2
GSM5590158 ATAC-seq_Wild-type_CD41_bioRep1_techRep1
GSM5590159 ATAC-seq_Wild-type_CD41_bioRep1_techRep2
GSM5590160 ATAC-seq_Wild-type_Flk1_bioRep1_techRep1
GSM5590161 ATAC-seq_Wild-type_Flk1_bioRep1_techRep2
GSM5590162 ATAC-seq_Wild-type_Flk1_bioRep2_techRep1
GSM5590163 ATAC-seq_Wild-type_Flk1_bioRep2_techRep2
GSM5590164 Bisulfite-seq_Wild-type_E14
GSM5590165 Bisulfite-seq_Wild-type_416B
GSM5590166 Bisulfite-seq_Wild-type_In-vitro-methylated-control
GSM5590167 416B_CTCF_ChIP-seq Rep1
GSM5590168 416B_CTCF_ChIP-seq Rep2

Genome browser session (e.g. UCSC)

A genome browser session has not been generated due to a limitation of the website being unable to display Tiled-C data at this time.

Methodology

Replicates

CTCF ChIP-seq in mESC: a single replicate of each clone was performed.
CTCF ChIP-seq in 416B cells: two ChIP-seq replicates were performed.

Sequencing depth

Samples were sequenced on Illumina NextSeq platform with paired end reads (40bp+40bp). Read depths are as follows:
CTCF_ChIP_P1_E7-1; 48715932 reads
CTCF_ChIP_P1_E7-2; 39459984 reads
CTCF_ChIP_P1_E9-1; 54806626 reads
CTCF_ChIP_P2_D10-1; 68159991 reads
CTCF_ChIP_P2_D2-1; 54941720 reads
CTCF_ChIP_P2_E9-1; 45106238 reads
CTCF_input_P1_E7-1; 95872376 reads
CTCF_input_P1_E7-2; 102846827 reads
CTCF_input_P1_E9-1; 109999245 reads
CTCF_input_P2_D10-1; 112310695 reads
CTCF_input_P2_D2-1; 111602425 reads
CTCF_input_P2_E9-1; 91847134 reads
416B_CTCF_rep1; 59608424 reads
416B_CTCF_rep2; 8964460 reads

Antibodies

anti-CTCF-unconjugated (EMD Millipore; 07-729)

Peak calling parameters

CTCF peaks were called using MACS2 with parameters -p 0.02 -f BAM -g mm using 416B input track as a control. In CTCF-KO mESC CTCF ChIP-seq, visual inspection was used to examine peaks.

Data quality

Sequencing data quality was assessed using the FASTQC tool. 44513 peaks were identified in 416B ChIP-seq with an FDR threshold below 0.02.

Software

MACS 2.0.10; meme 4.9.1_1; CCSeqBasic CM5; fastqc 0.10.1; trim_galore 0.3.1; deeptools 2.2.2

Flow Cytometry

Plots

Confirm that:

☒ The axis labels state the marker and fluorochrome used (e.g. CD4-FITC).

☒ The axis scales are clearly visible. Include numbers along axes only for bottom left plot of group (a 'group' is an analysis of identical markers).

☒ All plots are contour plots with outliers or pseudocolor plots.

☒ A numerical value for number of cells or percentage (with statistics) is provided.

Methodology

Sample preparation	Cells were isolated from differentiation cultures. Single cell suspensions were stained in FACS buffer (PBS 10% FCS) for 10-30 minutes on ice in the dark. Up to 10^6 cells were stained in 50ul buffer. After staining, antibody was diluted at least two-fold using FACS buffer, cells were centrifuged again, before resuspending in FACS buffer with the addition of Hoechst 33258 diluted 1:1000.
Instrument	FACS sorting was performed using a Fusion 2 Becton Dickinson machine.
Software	BD FACSDiva Software (version 8.0.1) was used to collect data. FlowJo (TreeStar, version 10.8) was used to analyse data.
Cell population abundance	Cell purity was over 90% in a post-sorted cell population re-run on the analyser.
Gating strategy	Initial gating was done to all samples the same irrespective of day of differentiation. This included cells being gated on FSC-A/SSC-A to identify cells. Cells were then gated on FSC-A/FSC-H to identify single cells. Cells were then gated on Hoescht negative to identify live cells. At day 4 of differentiation, cells were further gated on Flk1-APC positive cells. At day7 of differentiation, cells were further gated on Ter119-PE-Cy7 negative cells, and CD41-PE positive CD45-APC-eFluor780 negative cells.

☒ Tick this box to confirm that a figure exemplifying the gating strategy is provided in the Supplementary Information.
